# Micro-RNAs Shuttled by Extracellular Vesicles Secreted from Mesenchymal Stem Cells Dampen Astrocyte Pathological Activation and Support Neuroprotection in In-Vitro Models of ALS

**DOI:** 10.3390/cells11233923

**Published:** 2022-12-04

**Authors:** Francesca Provenzano, Sophie Nyberg, Debora Giunti, Carola Torazza, Benedetta Parodi, Tiziana Bonifacino, Cesare Usai, Nicole Kerlero de Rosbo, Marco Milanese, Antonio Uccelli, Pamela J. Shaw, Laura Ferraiuolo, Giambattista Bonanno

**Affiliations:** 1 Department of Pharmacy (DIFAR), University of Genoa, Viale Cembrano 4, 16148 Genova, Italy; 2 Sheffield Institute for Translational Neuroscience (SITraN), University of Sheffield, 385A Glossop Road, Sheffield S10 2HQ, UK; 3 Department of Neurosciences, Rehabilitation, Ophthalmology, Genetics, Maternal and Child Health (DINOGMI), University of Genoa, Largo Paolo Daneo, 316132 Genoa, Italy; 4 IRCCS Ospedale Policlinico San Martino, Largo Rosanna Benzi 10, 16132 Genoa, Italy; 5 Inter-University Center for the Promotion of the 3Rs Principles in Teaching & Research (Centro 3R), 56122 Pisa, Italy; 6 Institute of Biophysics, National Research Council (CNR), Via De Marini 6, 16149 Genoa, Italy; 7 TomaLab, Institute of Nanotechnology, National Research Council (CNR), Piazzale Aldo Moro 5, 0018 Rome, Italy

**Keywords:** amyotrophic lateral sclerosis, adult mice spinal cord astrocytes, iNPCs-derived human astrocytes, extracellular vesicles, mesenchymal stem cells, cell therapy

## Abstract

Amyotrophic lateral sclerosis (ALS) is a neurodegenerative disease with no effective cure. Astrocytes display a toxic phenotype in ALS and contribute to motoneuron (MN) degeneration. Modulating astrocytes’ neurotoxicity can reduce MN death. Our previous studies showed the beneficial effect of mesenchymal stem cell (MSC) administration in SOD1^G93A^ ALS mice, but the mechanisms are still unclear. We postulated that the effects could be mediated by extracellular vesicles (EVs) secreted by MSCs. We investigated, by immunohistochemical, molecular, and in vitro functional analyses, the activity of MSC-derived EVs on the pathological phenotype and neurotoxicity of astrocytes isolated from the spinal cord of symptomatic SOD1^G93A^ mice and human astrocytes (iAstrocytes) differentiated from inducible neural progenitor cells (iNPCs) of ALS patients. In vitro EV exposure rescued mouse and human ALS astrocytes’ neurotoxicity towards MNs. EVs significantly dampened the pathological phenotype and neuroinflammation in SOD1^G93A^ astrocytes. In iAstrocytes, exposure to EVs increased the antioxidant factor Nrf2 and reduced reactive oxygen species. We previously found nine miRNAs upregulated in MSC-derived EVs. Here, the transfection of SOD1^G93A^ astrocytes with single miRNA mimics reduced astrocytes’ activation and the expression of neuroinflammatory factors. Moreover, miR-466q and miR-467f mimics downregulate *Mapk11*, while miR-466m-5p and miR-466i-3p mimics promote the nuclear translocation of Nrf2. In iAstrocytes, transfection with miR-29b-3p mimic upregulated NQO1 antioxidant activity and reduced neurotoxicity towards MNs. MSC-derived EVs modulate astrocytes’ reactive phenotype and neurotoxicity through anti-inflammatory and antioxidant-shuttled miRNAs, thus representing a therapeutic strategy in ALS.

## 1. Introduction

Amyotrophic lateral sclerosis (ALS) is an adult-onset and fatal neurodegenerative disease characterised by upper and lower motor neuron (MN) cell death, producing muscle weakness and atrophy, paralysis, and finally neuromuscular respiratory failure [1,2]. Approximately 5–10% of ALS cases are familial [3] and about 20% of familial cases are due to mutations in the gene encoding Cu/Zn superoxide dismutase type 1 (SOD1). Indeed, transgenic SOD1 animal models, especially SOD1^G93A^ mice, show MN degeneration and ALS symptoms resembling the human disease [4,5].

ALS is a multifactorial and multicellular disease in which several mechanisms contribute to motor neuron injury [6,7,8,9]. In this scenario, the detrimental role of reactive astrocytes [10,11] in ALS [12,13,14] includes excitotoxicity [15,16], abnormal influx of Ca^2+^ into MNs [17], and altered astrocyte metabolism [18,19,20,21,22]. ALS astrocytes also release pro-inflammatory cytokines [23,24,25], which cause oxidative stress [26,27] and extracellular vesicles containing misfolded proteins [28,29]. Furthermore, ALS astrocytes show an aberrant content and transfer of miRNAs, affecting axonal and dendrite growth [30,31]. The number of dysregulated pathways make astrocytes key potential targets for neuroprotection in ALS.

No effective treatment is available for ALS [32,33], and attention has been devoted to innovative therapies, such as transplantation of mesenchymal stem cells (MSCs), derived from bone marrow or adipose tissue, which can differentiate in vitro into neuron-like cells and release neuroprotective factors [34]. Several studies have investigated the efficacy and safety of MSC administration in ALS patients [35,36,37,38].

Our previous work demonstrated that a single intravenous administration of mouse-bone-marrow-derived MSCs in SOD1^G93A^ mice extended lifespan, ameliorated motor performance, and reduced astrogliosis and inflammation in the spinal cord [39]. Since MSCs hardly reach the CNS [40,41], we hypothesised the involvement of paracrine mechanisms based on the release from MSCs of soluble factors and extracellular vesicles (EVs) containing proteins, messenger RNAs, and micro-RNAs (miRNAs) [42]. Indeed, MSCs release EVs containing miRNAs that can mitigate the inflammatory response [43,44,45].

Here, we demonstrate that MSC-derived EVs can ameliorate the toxic phenotype of mouse SOD1^G93A^ astrocytes and human-derived astrocytes (i-Astrocytes), harbouring SOD1 or C9*orf*72 mutations. We identified new miRNAs in MSC-derived EVs that ameliorate the toxicity of ALS astrocytes. Moreover, our results show that EV treatment of ALS astrocytes improves MN survival in astrocyte-MN co-cultures. These findings identify MSC-derived EVs and EV-shuttled miRNAs as possible mediators of the beneficial effects of MSCs.

## 2. Materials and Methods

### 2.1. Animals

B6SJL-TgN SOD1/G93A1Gur mice expressing a high copy number of mutant human SOD1 with a Gly93Ala substitution (SOD1^G93A^) and background-matched wild-type (WT) mice [46] were originally obtained from Jackson Laboratories (Bar Harbor, ME, USA) and bred at the Animal Facility of the Department of Pharmacy in Genoa, Italy, where they were kept until experiments were carried out. C57BL/6J mice, originally purchased from Charles River Italia (Milan, Italy) were maintained in our own colony at the Animal Facility of IRCCS Ospedale Policlinico San Martino, Genoa, Italy.

The SOD1^G93A^ mouse colony was maintained by crossing *SOD1^G93A^* male mice with background-matched B6SJL WT females, and selective breeding maintained each transgene in the hemizygous state. Mice carrying the *SOD1^G93A^* mutation were identified by analysing tissue extracts from tail tips as previously described [47]. Animals were housed (6/7 per cage) at constant temperature (22 ± 1 °C) and relative humidity (50%) with a regular 12 h light cycle (light 7 a.m.–7 p.m.). Food (type 4RF21 standard diet obtained from Mucedola, Settimo Milanese, Milan, Italy) and water were freely available. Both male and female mice were utilised and sexes were balanced in each experimental group to avoid bias due to intrinsic sex-related differences. For experimental use, animals were sacrificed at a late stage of the disease (about 120 days old) and scored according to motor impairment severity [39]. All efforts were made to minimize animal suffering and to use only the number of animals necessary to produce reliable results. For ethical issues related to the use of animals for experimental studies, refer to the “Institutional Review Board Statement” section.

### 2.2. Mesenchymal Stem Cell Culture and Extracellular Vesicle Isolation

MSCs were expanded from human and mouse bone marrow cells and characterized as previously described [48,49].

Human. Human bone marrow samples were collected (20 mL in sterile 50 mL tubes containing EDTA) by aspiration from the posterior iliac crests of healthy donors undergoing stem cell harvesting, at the Unità Operativa Ematologia, Ospedale Policlinico San Martino -IRCCS, Genoa, Italy. Mesenchymal stem cells were expanded from bone marrow cells and characterized as previously described [48]. Briefly, bone marrow mononuclear cells were isolated by density gradient centrifugation (1077 g/mL; Lympholyte Cell Separation Media; Cedar Lane) and seeded at the density of 25–30 × 10^6^ cells per 75 cm^2^ flask in Human MesenCult Basal Medium, added with its specific supplement (StemCell Technologies, Cambridge, UK) and incubated at 37 °C and 5% CO_2_. Bone marrow non-adherent cells were removed after 4 days, and culture medium was refreshed twice per week thereafter. At 80% confluence, cells were harvested with 0.05% trypsin and 0.02% EDTA (Euroclone, S.p.A, Milan, Italy) and plated in 75 cm^2^ flasks at the density of 7 × 10^5^ cells. Characterization of hMSC in culture was achieved after three passages in culture on the basis of the expression of the typical markers CD34-, CD45-, CD14-, CD73+, CD44+, CD105+ cells by flow cytometry, and on their ability to suppress T-cell proliferation.

A total of 85–90% confluent human MSCs were treated with interferon-γ 100 nM in serum-free media for 24 h in order to increase their immunomodulatory features [50]. At 20 min before extracellular vesicle (EV) isolation, 100 nM of adenosine 5′-triphosphate (ATP) was added to the media, followed by isolation of EVs (Exosome isolation kit, Thermo Fisher Scientific, Waltham, United States) according to the manufacturer’s instructions. Briefly, 5 mL of exosome isolation kit was added to 10 mL of media and left to incubate at 4 °C overnight, followed by transfer into ultracentrifugation tubes and centrifuging at 10,000× *g* for 60 min at 4 °C. The pellet containing the isolated fraction of exosome-enriched extracellular vehicles (EVs) was resuspended in RPMI and stored in 4 °C for up to 7 days before use for a cell culture experiment or in Qiazol for microarray analysis. Size distribution of EVs was evaluated with ZetaView (Particle Matrix, Meerbusch, Germany). Microarray analysis of miRNA in EVs was performed and analysed by LC Science (Houston, TX, USA) according to the MIAME guidelines [51].

Human bone marrow mesenchymal stem cells isolated from healthy donors were used up to a passage of P7, and grown in T75 flasks in Mesencult™ basal media with Mesencult™ stimulatory supplement (Stemcell Technologies, Cambridge, UK). Lyophilised Interferon-γ (R&D Systems) was reconstituted in dH_2_O. Cell-culture-grade adenosine 5′-triphosphate (ATP) disodium salt (CAS# 987-65-5, Merck Life Science UK Limited, Gillingham, UK) was reconstituted in sterile dH_2_O. A total of 85–90% confluent human MSCs were treated with interferon-γ 100 nM in serum-free media for 24 h. At 20 min before EV isolation, 100 nM of adenosine 5′-triphosphate (ATP) was added to the media, followed by isolation of EVs (Exosome isolation kit, Thermo Fisher Scientific, Waltham, United States) according to the manufacturer’s instructions. Briefly, 5 mL of exosome isolation kit was added to 10 mL of media and left to incubate at 4 °C overnight, followed by transfer into ultracentrifugation tubes and centrifuging at 10,000× *g* for 60 min at 4 °C. EVs were stored in 4 °C for up to 7 days before use. Size distribution of EVs were evaluated with ZetaView (Particle Matrix, Meerbusch, Germany).

Mouse. Bone-marrow-derived MSCs were isolated from 6–8-week-old C57BL/6J mice, expanded in serum-free murine Mesencult medium (Stem Cell Technology, Cambridge, UK), and characterized as described previously [49]. Expanded MSCs were stimulated at passage 14/15 with 10 ng/mL interferon-γ(IFN-γ) for 24 h at 37 °C in serum-free RPMI-based medium in order to increase their immunomodulatory features, as demonstrated by their ability to inhibit T-cell proliferation [50]. To increase the production and secretion of EVs, expanded IFN-γ-primed and unprimed MSCs were stimulated for 20 min with 1 mM adenosine triphosphate (ATP, Merck, Milan, Italy) in serum-free RPMI-based medium at 37 °C [52]. The resulting supernatant was centrifuged at 2000× *g* at 4 °C for 20 min to eliminate cells and debris and incubated overnight at 4 °C with 0.5 volume of Total Exosome Isolation Kit (Thermo Fisher Scientific, Monza, Italy). The sample was centrifuged at 10,000× *g* at 4 °C for 1 h and the pellet containing the exosome-enriched EV isolated fraction was resuspended according to the experimental needs.

EVs were previously characterized by Western blotting, contrast phase microscopy and electron microscopy [45].

### 2.3. In Vitro Treatment with Extracellular Vesicles

After EV isolation, MSCs were detached with Trypsin-EDTA 1X and counted by a Neubayer Chamber to set up a standard protocol for the number of EVs to be added to the astrocyte media during in vitro treatment. In each experiment, the ratio of 1:6 between cultured astrocytes and EV-generating MSCs was maintained for in vitro treatments. Accordingly, we used a number of EVs generated by 6 × 10^5^ or 2 × 10^4^ MSCs for the treatment of astrocytes plated on 35 mm Petri dishes or 24-well plates, respectively.

Mouse SOD1^G93A^ astrocytes and iAstrocytes were treated with MSC-derived EVs in DMEM without FBS or knockout serum, respectively. After 24 h treatment, astrocytes were collected, pelleted, and stored for WB and RT-qPCR experiments, or fixed with paraformaldehyde (PFA) 4% in PBS for immunofluorescence. For MN viability experiments, the medium of mouse or human astrocytes was removed, and MNs were seeded on confluent astrocytes previously treated (24 h) or not with EVs.

### 2.4. Mouse Spinal Cord Primary Astrocyte Cell Culture Preparation

Spinal cord astrocyte primary cell cultures were prepared from late symptomatic (120-day-old) adult SOD1^G93A^ and age-matched WT mice as previously described [53,54]. In detail, late symptomatic (120-day-old) SOD1^G93A^ mice and age-matched WT mice were euthanized by cervical dislocation by well-trained personnel and spinal cords were rapidly removed. The dissected tissue was mechanically chopped with a scalpel and the chopped spinal cord was dispersed and further fragmented in Dulbecco’s Modified Eagle Medium (DMEM; Euroclone, S.p.A, Milan, Italy, Cat# ECM0728L) containing 10% Fetal Bovine Serum (Euroclone, Cat# ECS0180L), 1% glutamine (Euroclone, S.p.A, Milan, Italy Cat# ECB3004D) and 1% Penicillin/Streptomycin (Euroclone, Cat# ECB3001D); then, the preparation was seeded at the optimal density in two 35 mm Petri dishes, pre-coated with poly-L-ornithine hydrochloride (1.5 µg/mL; Merck, Milan, Italy, Cat# P2533) and laminin (3 µg/mL; Merck, Milan, Italy, Cat# L2020). The preparation was placed at 37 °C in humidified 5% CO_2_ incubator; then, after 5 days, the medium containing tissue fragments was replaced with fresh complete DMEM. After 7DIV, adherent astrocytes were detached by Trypsin-EDTA 1× (Euroclone, S.p.A, Milan, Italy, Cat# ECB3052B) and re-plated on pre-coated 35 mm Petri dishes to obtain the homogeneous confluence at the optimal density of 10^5^ cells. The purity of spinal cord adult astrocyte cell cultures was checked with flow cytometry and immunofluorescence as previously described [54]. For immunofluorescence (IF) analyses, astrocytes were also re-plated on pre-coated glass coverslips placed at the bottom of 24-multiwell plates, at a density of 3 × 10^4^ cells per well. After 20DIV, astrocytes were treated for 24 h with Evs or transfected for 48 h with synthetic mimics; after treatment, astrocytes were finally detached and collected for Western blot and RT-qPCR experiments, or fixed with 4% PFA (Merck, Milan, Italy,, Cat# 47608) for IF studies.

### 2.5. Mouse Spinal Cord Motor Neuron Preparation and MN/Astrocyte Co-Cultures

MNs were isolated from the spinal cord of SOD1^G93A^ E13.5 mouse embryos as previously described with some modifications [55]. Briefly, the spinal cord was isolated from embryos by microscopy dissection, then meninges and the dorsal root ganglia were removed. The tissue was digested with 0.5% trypsin (Merck, Milan, Italy, Cat# T4799) in Hank’s Balance Salt Solution (HBSS) for 20 min at 37 °C. Then, trypsin solution was replaced with a mix of 0.4% BSA (Merck, Milan, Italy, Cat# A3311) in Leibovitz-15 medium (Merck, Milan, Italy, Cat# L5520) and 0.02 mg/mL Deoxyribonuclease I (DNAse; Merck, Milan, Italy, Cat#DN25) and gently triturated. The tissue homogenate was stratified on 6.2% OptiPrep (Merck, Milan, Italy,, Cat# D1556) cushion and centrifuged 500× *g* for 15 min at room temperature. After centrifuging, the MN-enriched cell population was localized at the interface between the OptiPrep solution and the medium. The MN band was collected and re-suspended in MN medium, composed of neurobasal medium (Thermo Fisher Scientific, Monza, Italy, Cat# 21103-049), 2% B27 supplement (Thermo Fisher Scientific, Monza, Italy, Cat# 17504044), 2% horse serum (Thermo Fisher Scientific, Monza, Italy, Cat# 16050130), 0.5 mM stable L-Glutamine (Thermo Fisher Scientific, Monza, Italy, Cat#35050038), 25 µM Mercapto ethanol (Sigma-Aldrich, Cat# M6250), 10 ng/mL ciliary neurotrophic factor (CNTF; Merck, Milan, Italy, Cat# C3835), 100 pg/mL glial-derived neurotrophic factor (GDNF; Merck, Milan, Italy, Cat# G1401), and 5 µg/mL Penicillin/Streptomycin. MN suspensions were centrifuged at 75× *g* for 20 min and the pellet was suspended in 1 mL of MN medium plus 50 µL of Chick Embryo Extract (US Biological, Salem, Massachusetts, United States, Cat# C3999). MNs were seeded at a density of 5 × 10^4^ MNs in a 35 mm Petri dish on confluent adult astrocytes prepared from 120-day-old SOD1^G93A^ mice, previously treated or not with EVs. Confocal microscopy representative images showing MN/astrocyte co-cultures labelled with specific markers for astrocytes and MNs are reported in Supplementary Figure S1 (panels a–d). After 3 days, and then three times a week, the medium was replaced with fresh MN medium. To assess MN viability, MN were counted in an area equal to 1 cm^2^ (exploiting a 10 × 10 mm grid pre-designed at the bottom of the Petri dish) from day 4 after seeding. The average number of MNs at the count, starting from day 4, in a 1 cm^2^ square area, was around 700 ± 200 MNs. The number of MNs was recorded three times a week for 14 days. The percentage of surviving MNs at each time-point was calculated as % vs. the total number of MNs, counted in the same 1 cm^2^ square area of the respective dish, at the day 4 of co-culture (starting day of the cell count experiment, reported as 100% of total MNs).

### 2.6. iAstrocytes Preparation and iAstrocyte/MN Co-Cultures

Induced neural progenitor cells (iNPCs) were reprogrammed from the fibroblast of ALS patients (C9orf72 patients reported as C9, or patients carrying the SOD1^A4V^ mutation, reported as SOD1) or control donors (reported as CTR), as previously reported [56], and banked. The details of the donors are listed in Supplementary Table S1.

iNPCs were grown on fibronectin-coated plates and were fully differentiated into iAstrocytes on day 7 after plating. Immunofluorescence representative images showing iAstrocytes differentiated from iNPCs, of healthy donors and ALS patients, stained for cell identity markers, are reported in Supplementary Figure S2 (panels a,b). iAstrocytes were maintained in 37 °C and 5% CO_2_ in DMEM (Fisher Scientific, Milan, Italy) supplemented with 10% fetal bovine serum (FBS) (Life science production), and 0.2% N-2 (Gibco).

Hb9-GFP-positive motor neurons were prepared from mouse embryonic stem cells (mESC) containing a GFP gene under the Hb9 motor neuron promoter, a kind gift from Prof. Thomas Jessell (Columbia University, New York, NY, USA). Hb9-GFP mESCs were maintained on a mouse embryonic fibroblast (Merck, Burlington, MA, USA) feeder layer in mESC media (KnockOut-DMEM, 15% (*v*/*v*) embryonic stem cell FBS, 2mML-glutamine, 1% (*v*/*v*) nonessential amino acids (from Thermo Fisher Scientific, Waltham, United States) and 0.00072% (*v*/*v*) β-mercaptoethanol (Merck, Milan, Italy). mESCs were then differentiated into MN-enriched cultures via embryoid bodies (EBs). Briefly, mESCs were lifted with trypsin, resuspended in EB medium (DMEM/F12), 10% (*v*/*v*) knockout serum replacement, 1% N_2_, 1 mM L-glutamine (from Thermo Fisher Scientific, Waltham, United States), 0.5% (*w*/*v*) glucose and 0.0016% (*v*/*v*) β-mercaptoethanol and seeded into non-adherent Petri dishes. EB media was replenished every day, supplemented with 2 μM retinoic acid and 1 µM Smoothened agonist (from Merck, Milan, Italy) to induce motor neuron differentiation.

For co-culture, day 5 iAstrocytes were re-seeded in Greiner 96-wellplates at a density of 10,000 cells/well, followed by addition of treatment (EVs or miRNA) on day 6 in serum-free media. On day 7, EBs were dissociated with 200 U/mL papain (Merck, Milan, Italy) and GFP+ neurons were seeded into 96-wellplates containing iAstrocytes. Immunofluorescence representative images showing iAstrocytes/MNs co-cultures, stained for cell identity markers, are reported in Supplementary Figure S2 (panel c). For experiments assessing motor neuron survival, co-culture plates were imaged on days 1 and 3 using the InCell high content microscope and the number of Hb9-GFP+ neurons with axons was quantified in the whole well. Images obtained with InCell were used for semi-automated high-throughput analysis with Columbus microscopy software [57]. The percentage of Hb9-GFP+ neurons with axons was calculated as % of the total number of MNs at day 1 over day 3. Typically, MN survival at day 3 on healthy control iAstrocytes was between 65–75%, while on control astrocytes we typically observed a decrease between 50–70% depending on the toxicity of the individual ALS donor. To reduce inter-week variation, the data were normalised to the average of the healthy control co-culture in the same 96-well plate = 100%. Each iAstrocyte line was assessed in 3 independent experiments (i.e., from 3 independent iNPC differentiations at different passages) and 3 technical replicates were run in each experiment.

### 2.7. Immunofluorescence Experiments

Adult astrocytes cultured from SOD1^G93A^ (120-day-old) and age-matched WT mice, seeded on 12 mm diameter glass coverslips, at the bottom of 24-multiwell plates, were fixed with 4% PFA (Merck, Milan, Italy, Cat# 47608). After fixing, cells were permeabilized with methanol for 5 min at −20 °C. Bovine serum albumin (BSA) 0.5% in PBS, for 15 min at room temperature, was used to arrest the process. All primary antibodies were diluted in 3% PBS-BSA blocking solution. Primary antibodies were incubated overnight at 4 °C (Supplementary Table S3). The day after, cells were washed in 0.5% PBS-BSA before the incubation of 1 h at room temperature with the secondary antibody (Supplementary Table S3). Secondary antibodies were diluted 1:3000 in PBS containing 3% BSA. Then, cells were washed in PBS and the coverslip was assembled on a microscopy glass slide through Fluoroshield^TM^ with DAPI (Merck, Milan, Italy, Cat# F6057). Fluorescence image (512 × 512 × 8 bit) acquisition was performed by a three-channel Leica TCS SP5 laser-scanning confocal microscope equipped with 458, 476, 488, 514, 543 and 633 nm excitation lines through a plan-apochromatic oil immersion objective 63×/1.4. Light collection was optimized according to the combination of the chosen fluorochromes, and sequential channel acquisition was performed to avoid crosstalk. The Leica “LAS AF” software package was used for image acquisition. The quantitative analyses related to immunofluorescence confocal microscopy studies were performed by calculating the co-localization coefficients of the proteins of interest, according to Manders’ and Costes’ theories [58,59], thus allowing a direct quantitative correlation between the intensity of co-localization of the protein of interest with respect to a stable housekeeping protein. Quantitative results were expressed as the relative co-localization intensity of the protein of interest, respect to the stable reference housekeeping protein 3-phosphate dehydrogenase glyceraldehyde (GAPDH). All the immunofluorescence studies were assessed by performing at least 3 independent experiments, run in triplicate (i.e., 3 wells per experiment). Each acquired image included an average of at least 6 to 12 cells homogenously distributed, thus resulting in 50 to 100 assessed cells for each sample, in each experiment.

To check MN/astrocyte co-culture purity (see Supplementary Figure S1), the cells were seeded on 12 mm diameter glass coverslips at the bottom of 24-multiwell plates; after 8 days of co-culture, cells were fixed with 4% PFA (Merck, Milan, Italy, Cat# 47608). After fixing, cells were permeabilized with methanol for 5 min at −20 °C. Bovine serum albumin (BSA) 0.5% in PBS, for 15 min at room temperature, was used to arrest the process and saturate the non-specific binding sites. MN/astrocyte co-cultures were stained with cell-specific primary antibodies for astrocytes (rabbit anti-GFAP plyclonal antibody, Merck, Milan, Italy, # HPA056030, 1:1000) and motor neurons (chicken anti-beta tubulin III polyclonal antibody, Abcam #ab41489, 1:1000; mouse anti-ChAT monoclonal antibody, Merck, Milan, Italy #AMAB91130, 1:500; rabbit anti-HB9 polyclonal antibody, Thermo Fisher #PA5-23407, 1:500; rabbit anti-Islet1 polyclonal antibody Abcam #ab20670, 1:500). All primary antibodies were diluted in 3% PBS-BSA blocking solution. Primary antibodies were incubated overnight at 4 °C. The day after, cells were washed in 0.5% PBS-BSA before incubation of 1 h at room temperature with the secondary antibody conjugated with specific fluorophores. Secondary antibodies were diluted 1:3000 in PBS containing 3% BSA. Then, cells were washed in PBS and the coverslip was assembled on a microscopy glass slide through Fluoroshield^TM^ (Merck, Milan, Italy, Cat# F6057). Confocal microscopy fluorescence images were acquired as described above for spinal cord astrocyte primary cultures.

### 2.8. Western Blot

Human. On day 5, iAstrocytes were re-seeded into 6-well plates at 250,000 cells/well, treated with EVs in serum free media on day 6, and full media was replaced 24 **h** later to allow cells to recover for another 24 h. Cells were scraped on day 7, pelleted and lysed on ice with immunoprecipitation lysis buffer (150 mM NaCl, 50 mM HEPES, 1 mM EDTA, 1 mM DTT, 0.5% (*v*/*v*) Triton X-100, pH 8.0) with protease inhibitor cocktail for 15 min. Lysates were centrifuged at 17,000× *g* for 5 min at 4 °C, and supernatants quantified for protein content with Bradford assay measured with a WPA S1200 Diode Array Spectrophotometer (Biochrom, Nottingham, UK). Protein was diluted in 4X Laemmli buffer (228 mM Tris-HCl, 38% (*v*/*v*) glycerol, 277 mM SDS, 0.038% (*w*/*v*) bromophenol blue, 5% (*v*/*v*) β-mercaptoethanol, pH 6.8) and immunoprecipitation buffer, and samples were boiled at 93 °C for 5 min. A total of 15 μg of protein or 2 μL of Blu-Eye pre-stained ladder was loaded in each well of 12% SDS-acrylamide gels, and gels were run at 120V using a mini-PROTEAN Tetra Handcast systems (Bio-Rad, Hercules, California, USA). Semi-dry transfer was completed onto a nitrocellulose membrane, in transfer buffer (47.9 mM Tris, 38.6 mM glycine, 1.38 mM SDS, 20% (*v*/*v*) methanol) in a semi-dry transfer apparatus applying 0.15 A per membrane for 1 h at room temperature. Membranes were blocked in 5% milk in Tris-buffered saline/0.1% Tween-20 (TBST) for 30 min, before incubating in primary antibodies NLRP3 (1:100, Abcam, Cambridge, UK, Cat# ab210491) and p-p65 (1:200, Cell Signalling, Leiden, The Netherlands, Cat#3033S) in 5% milk/TBSTat 4 °C overnight on rollers. Anti-rabbit or anti-mouse-HRP secondary antibodies (Promega, Milan, Italy, W4011 and W4021, respectively) were diluted 1:5000 in 5% milk/TBST and incubated with the membrane in room temperature for 60 min under agitation. Membranes were incubated in enhanced chemiluminescence (ECL) developer (Pierce) for 30 s before imaging with the Gbox imaging system (Syngene, Karnataka, India). Protein expression was quantified by band densitometry using GeneTools software (Syngene, Karnataka, India) and normalised to GAPDH as a loading control.

Mouse. After 20DIV, mouse astrocytes were treated with MSC-derived EVs for 24 h. Then, samples for Western blot analysis were obtained by detaching cells with Accutase^®^ (Euroclone, S.p.A, Milan, Italy, Cat# ECB3056D) and suspending them in PBS. Cells were centrifuged at 17,000× *g* for 1 min and PBS was removed. Pellets were lysed on ice with immunoprecipitation buffer [150 mM NaCl, 50 mM HEPES, 1 mM EDTA, 1 mM DDT, 0.5% (*v*/*v*) Triton X-100, pH 8.0] containing a protease inhibitor cocktail for 15 min. Lysates were centrifuged at 17,000× *g* for 5 min and supernatants quantified for protein content by Pierce^TM^ BCA protein assay (Thermo Fisher Scientific, Waltham, United States, Cat#23227) according to the manufacturer’s protocol. An appropriate amount (15 μg) of total proteins were separated by SDS-polyacrylamide gel electrophoresis using 4–20% precast gels (Bio-Rad, Milan, Italy, Cat# 4568094) and transferred to nitrocellulose membranes saturated for non-specific membrane binding sites using 5% skimmed-milk solution. Membranes were incubated overnight at 4 °C with the following primary antibodies: rabbit polyclonal anti-GFAP (1:1000, Merck, Milan, Italy, Cat# G4546), mouse monoclonal anti-Vimentin antibody (1:1000; Merck, Milan, Italy, Cat# V2258), mouse monoclonal anti-S100β antibody (1:100; Merck, Milan, Italy, Cat# MAB079-1), rabbit recombinant monoclonal anti-NLRP3 antibody (1:100, Abcam, Cambridge, UK, Cat# ab210491), rabbit polyclonal anti-Nrf2 antibody (1:500, Abcam, Cambridge, UK, Cat# ab31163), rabbit polyclonal anti-NQO1 antibody (1:1000, Abcam, Cambridge, UK, Cat# ab34173), and mouse monoclonal anti-GAPDH antibody (1:1000, Merck, Milan, Italy, Cat# G8795). The day after, membranes were incubated for 1 h at room temperature with anti-rabbit or anti-mouse-HRP secondary antibodies (Merck, Milan, Italy, Cat# A9169 and A9044, respectively) and diluted 1:5000 in 5% skimmed-milk solution. Membranes were then exposed to enhanced chemiluminescence developer (ECL-SuperSignalTM West Femto Maximum Sensitivity Substrate, Thermo Fisher Scientific, Waltham, United States, Cat# 34095] for 60 s. Bands were detected and analysed for optical density using an enhanced chemiluminescence system (Alliance 6.7 WL 20 M, UVITEC, Cambridge, UK) and UV1D software (UVITEC). Bands of interest were normalized for GAPDH level in the same membrane. The concentration of proteins felt in the linear portion of the curve.

### 2.9. ELISAs and Cytokine Multiplexing

Human. iAstrocytes in monoculture were treated with EVs in serum-free media on day 6, followed by washing and replacing with full media on day 7 and collecting media and pelleting cells on day 8. Media was centrifuged at 2000× *g* for 5 min to pellet debris, and the supernatant was aliquoted and snap-frozen in liquid N_2_ and stored at −80 °C until use. Human TNF-α and IL-1β Quantikine^®^ ELISA kits were purchased (R&D systems, Minneapolis, Canada, United States) and used according to manufacturer’s instructions. Plates were read using a Pherastar spectrophotometer. For multiplex measurement of IL-6 and CCL-2, BD™ Cytometric Bead Array (CBA) kits were used according to manufacturer’s instructions.

Mouse. Mouse astrocytes, cultured in 35 mm Petri dishes, were treated with MSC-derived EVs for 24 h in serum-free DMEM. DMEM containing EVs was replaced with complete fresh DMEM, and astrocyte-conditioned medium was collected 24 h after EVs withdraw. Astrocyte-conditioned medium was centrifuged 2000× *g* for 5 min. Then, IL-1β, TNF-α, IL-6 and CCL2 concentrations were measured with a specific enzyme-linked immunosorbent assay (ELISA) kit (R&D Systems, Minneapolis, Canada, United States Cat# DY401, DY410, DY406 and DY479, respectively) according to the manufacturer’s protocol.

### 2.10. Astrocyte Transfection with miRNA Mimics and Quantitative RT-PCR

Human and mouse synthetic miRNA mimics were used to transfect (for 48 h) iAstrocytes or SOD1^G93A^ mouse spinal cord astrocytes using the HiPerFect^®^ Transfection Reagent (Qiagen, Manchester, UK), according to the manufacturer’s instructions. The list and sequence of the synthetic mimics are reported in Supplementary Table S2.

Human. Synthetic miRNA mimic hsa-miR-29b3P was obtained from Ambion^®^ Pre-miR™ (ThermoFisher, Waltham, United States,). The miRNA mimic was resuspended in DEPC-treated water and stored at −20 °C until further use. On day 6, iAstrocytes were treated with synthetic miRNA by adding the miRNA mimic to serum-free DMEM to a final concentration of 50 nM, along with transfection reagent HiPerfect (Qiagen, Waltham, United States,) used according to manufacturer’s instructions. The mixture of media, miRNA and HiPerfect was briefly vortexed, allowed to incubate for 5–10 min at room temperature, and added onto the cells drop-wise. Full media was added onto the cells 6 h later, and cells were kept at 37 °C until the endpoint 24 h after transfection. For miRNA transfection validation, RNA was extracted with a Direct-zol RNA isolation kit (Zymo Research, Cambridge, UK) and miRNA content quantified with the small RNA kit in Bioanalyser (Agilent, Technologies LDA UK Limited, Cheshire, UK). For quantification of downstream genes with RT-qPCR, RNA was extracted from *n =* 3 independent experiments with the RNeasy kit (Qiagen) and concentration were quantified with Nanodrop. A total of 400 ng of RNA was converted to cDNA with the High-Capacity cDNA Reverse Transcription Kit (Thermo Fisher Scientific, Waltham, United States) in a Stratagene thermocycler. cDNA was stored at −20 °C until use. SYBR Green qPCR Master Mix (Low ROX-Bimake, Munich, Germany, Cat# B21702) was pipetted in low profile 96-wellplates, loading 20 ng of cDNA and 5 µM of forward and reverse primers (Merck, Life Science UK Limited, Gillingham, UK) in technical replicates. Plates were loaded onto a CFX 96TMReal-Time System (Bio-Rad) and fluorescence for SYBR was measured. Quantitative RT-PCR data were analysed using CFX Manager 3.1 (Bio-Rad, Hercules, California, USA) GraphPad Prism and Sigma Plot/Sigma Stat software.

Mouse. In total, 7 × 10^4^ or 2 × 10^5^ astrocytes were plated in 24-well plates or 35 mm-Petri dishes, respectively, in DMEM w/o FBS and transfected using the HiPerFect^®^ Transfection Reagent (Qiagen, Milan, Italy), according to the manufacturer’s instructions, with mimics specific for miRNA (miRNA Mimic miRNA, Qiagen, Milan, Italy) and with MISSION miRNA Mimic Negative Control (Merck, Milan, Italy,), a synthetic miRNA which does not recognize any mRNA target in cells (Cneg), and with iBONi siRNA positive control-P4M (Riboxx), which inhibits the translation of GAPDH in cells, as an indicator of efficient transfection. After 48 h incubation at 37 °C in humidified 5% CO_2_, astrocytes were fixed with 4% PFA for confocal microscopy or were detached with Accutase^®^ (Euroclone S.p.A, Milan, Italy, Cat# ECB3056D) and the cell pellet re-suspended in 500 μL of QIAzol Lysis reagent (Qiagen, Milan, Italy, Cat# 79306) for qPCR experiments. The sequence of mimics used are summarised in Supplementary Table S2. Total RNA was isolated from astrocytes using QIAzol Lysis Reagent according to the manufacturer’s instructions. First-strand cDNA was synthesized with 500 ng of total RNA from primary astrocytes using QuantiTect Reverse Transcription kit (Qiagen, Milan, Italy), in a final volume of 20 μL. Real-time polymerase chain reaction (RT-PCR) was performed in LightCycler 480 (Roche, Monza, Italy) in duplicate in a final volume of 20 μL containing 50 ng cDNA, 1 μL of each primer pair 20 μM (TIB MolBiol, Genoa, Italy), 10 μL of FastStart Essential DNA Green Master Mix (Roche). The amplification of the GAPDH as housekeeping gene was adopted to normalize expression data. Primer sequences used: mitogen-activated protein kinase (MAPK) kinase kinase 8 (Map3k8) forward (5′-TTCCAGTGCTCATGTACTCCA-3′) and reverse (5′-GGACTGCTGAACTCTGTTTGC-3′); MAPK-activated protein kinase 2 (Mk2) forward (5′-AGTGCAGCTCCACCTCTCTG-3′) and reverse (5′-CAGCAAAAATTCGCCCTAAA-3′); Mitogen-Activated Protein Kinase 11 (MAPK11) forward (5′-CACTGCTGAGGTCCTTCTGG-3′) and reverse (5′-CCTGAGGTTCTGGCAAAGAT-3′) and GAPDH forward (5′-ATGGTGAAGGTCGGTGTGA-3′) and reverse (5′-AATCTCCACTTTGCCACTGC-3′).

### 2.11. CellROX Reactive Oxygen Species Probe

Human iAstrocytes were re-seeded in black clear-bottom 96-well plates (Greiner) on day 5. On day 6, cells were incubated with Evs in serum-free DMEM for 24 h at 37 °C, followed by washing out EVs and replacing with full-serum DMEM for 24 h. On day 8, CellROX^®^ Orange (Thermo Fisher Scientific, Waltham, United States, Em 565 nm) was added to a final concentration of 5 μM at 37 °C for 30 min; the cells were washed with PBS and the medium replaced with phenol-red-free DMEM media. Live cells were imaged with an Opera Phenix high-content imager (PerkinElmer) and analysed with the Columbus Image Data Storage and Analysis System™ version 2.8 (PerkinElmer, Buckinghamshire, UK). The software allows for the generation of a cell mask that separates individual cells and calculates fluorescence intensity against the background signal. Fluorescence intensity per pixel in each well was calculated and these values were normalised to the average of the 2 wells containing untreated healthy control fluorescence intensity, set as 100 in each experiment. All samples were assessed in 3 independent experiments in duplicate (i.e., 2 wells per experiment). Whole-well image analysis was performed in each experiment, thus resulting in >1000 cells assessed for each sample in each experiment per cell donor.

### 2.12. Immunofluorescence Quantification of Nrf2 and NQO1

Mouse. Mouse-derived spinal cord astrocytes cultured from adult SOD1^G93A^ (120-day-old) and age-matched WT mice, were seeded on 12 mm diameter glass coverslips, at the bottom of 24-multiwell plates, treated or not with MSC-derived EVs or miRNA mimics, were fixed and stained with specific primary and secondary antibodies as detailed above to study Nrf2 cellular-specific localization (nuclear/cytoplasm ratio). The nuclear Nrf2 signal was quantified using DAPI as a regional mask to separate nucleus from cytoplasm regions, then the Nrf2 signals, subtracting the background signal outside the cells, were selectively quantified in these two regions, and the ratio between Nrf2 (nucleus) over Nrf2 (cytosol) fluorescence intensity was calculated and reported as quantitative analyses.

Human. On day 6, iAstrocytes in 96-well plates were treated with EVs for 24 h followed by a 24 h wash-out step, as described in previous sections. Cells were fixed at day 8 with 4% paraformaldehyde for 10 min, followed by PBS washing and blocking with 5% donkey serum in 0.05% Triton-X/PBS for 30 min. Anti-nuclear factor erythroid 2–related factor 2 (Nrf2) (rabbit polyclonal, Abcam ab31163) and goat anti-NAD(P)H Quinone Dehydrogenase 1 (NQO1) (Abcam ab2346) antibodies were added at a dilution of 1:200 in 1% donkey serum/0.05% Triton-X/PBS and the cells were incubated overnight at 4 °C, washed with PBS, and incubated with goat anti-rabbit-488 (Abcam, Cambridge, UK, ab150077) and donkey anti-goat-555 (Abcam, Cambridge, UK, ab175704) antibodies, diluted at 1:500 at room temperature for 60 min. Cells were washed and stained with Hoechst 33342 (MedChem Express LCC, Monmouth Junction, United States) and imaged with an Opera Phenix high content imager. Images were analysed with Columbus software, in which cell masks were created and fluorescence intensity was quantified in the nuclei and cytoplasm. Immunofluorescence data were normalised per cell and reported as % of the untreated healthy control. Nuclear Nrf2 signal was quantified using DAPI as a regional mask, while NQO1 was quantified in the cytoplasm excluding the nuclear area. As described above, >1000 cells for each sample per experiment, per cell donor, were assessed.

### 2.13. Statistics

The Sigma Stat Software (Version 3.5 2006; Inc., San Jose, CA, USA; RRID:SCR_003210) was used for statistical analysis and the Sigma Plot Software (Version 10.0- 006, Inc., San Jose, CA, USA; RRID:SCR_010285) for figure plots. All experiments were performed with a minimum of three independent biological replicates. The number of biological and technical replicates, statistical tests used, and *p* values are reported in the figure legends. The threshold for statistical significance (P) was set at *p* < 0.05. Data are always presented as mean ± standard error of mean (SEM).

## 3. Results

### 3.1. MSC-Derived EVs Reduce the Toxicity of Mouse- and Human-Derived ALS Astrocytes towards MNs

Mouse- or human-bone-marrow-derived MSCs were treated with interferon-γ (IFN-γ; 24 h) to activate their immunosuppressive and neuroprotective functions [60]. We have previously detected the morphology and purity of mouse-MSC-derived EVs [45]. The human-MSC (hMSCs)-derived EVs, in resting conditions and after IFN-γ priming, displayed typical vesicle biogenesis markers that increased, albeit not significantly, after exposure to IFN-γ (Figure S1a). The size distribution analysis produced prominent peaks between 100 and 175 nm, confirming the achievement of high-concentration EV preparations (Figure S1b).

**Figure 1:**
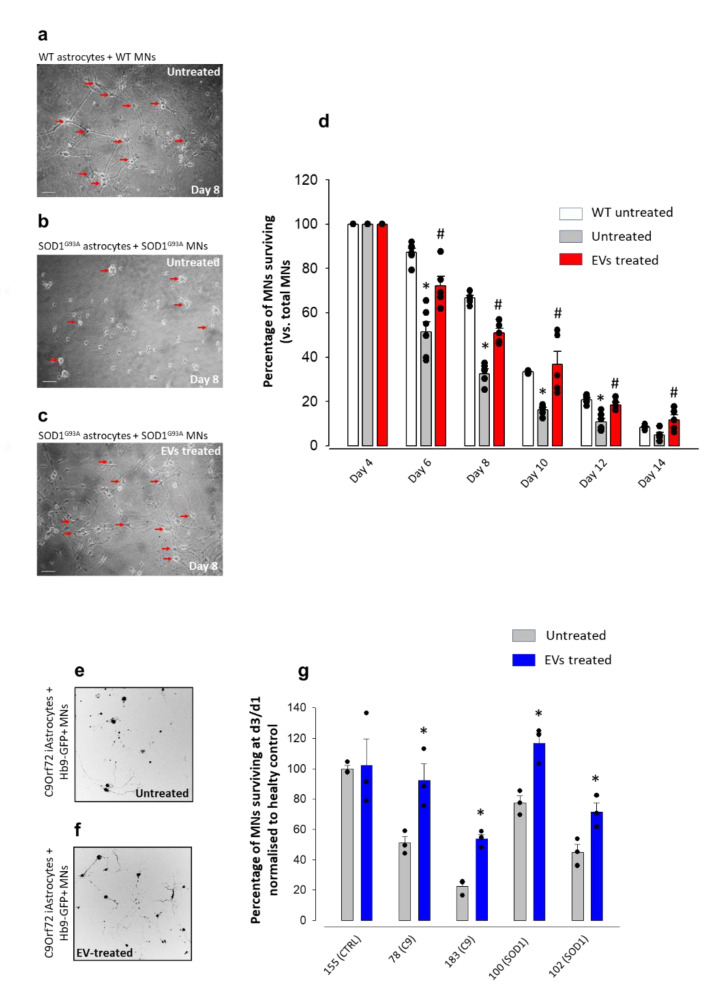
Astrocyte toxicity to motor neurons is reduced when astrocytes are pre-treated with MSC-derived EVs. (**a**–**d**) Viability of motor neurons (MNs) co-cultured with WT astrocytes, untreated (**b**) and EV-treated (**c**) spinal cord astrocytes from adult SOD1^G93A^ mice. (**a**,**c**) Representative phase-contrast microscopy images (100×) of MNs at day 8 of co-culture with WT astrocytes (**a**), untreated SOD1^G93A^ astrocytes (**b**) or with SOD1^G93A^ astrocytes pre-treated for 24 h with EVs (**c**); scale bar: 50 µm. (**d**) Quantification of MN viability expressed as percent (%) MN survival (calculated with respect to the total number of MN at day 4 in vitro -4DIV) when co-cultured with WT astrocytes, untreated or EV-treated SOD1^G93A^ astrocytes. Data represent the means ± SEM of *n* = 5–6 independent experiments. Statistical significance for *p* < 0.05 at least (* vs. WT and # vs. MNs + untreated SOD1^G93A^ astrocytes; one-way ANOVA, followed by Bonferroni post hoc test). (**e**–**g**) Viability of MNs co-cultured with untreated and EV-treated patient-derived reprogrammed astrocytes (iAstrocytes). (**e**,**f**) Representative InCell images of MNs after 3 days of co-culture with iAstrocytes, untreated (**e**) or treated (**f**) with human-MSC-derived EVs. (**g**) Quantification of MN survival expressed as the percentage of MNs with axon (calculated with respect to the total number of MNs at day 1) after 3 days of co-culture with iAstrocytes untreated or pre-treated with EVs. Data represent the means ± SEM of *n* = 3 independent experiments. Statistical significance (*) vs. untreated conditions for *p* < 0.05 at least (two-tailed Student’s t-test). t = −2.167, *p* = 0.046 vs. 78(C9) untreated iAstrocytes; t = −3.393, *p* = 0.009 vs. 183(C9) untreated iAstrocytes.

We set up mouse primary astrocyte-MN co-cultures to determine whether EVs have a neuroprotective effect on ALS MNs. We pre-treated adult SOD1^G93A^ astrocytes with MSC-derived EVs for 24 h; after wash-out, we seeded WT or SOD1^G93A^ mouse embryonic spinal cord MNs on WT untreated, SOD1^G93A^ EV-treated or SOD1^G93A^ untreated astrocytes (Figure 1a–c) and assessed the viability of MNs over 4–14 days of co-culture (Figure 1d). On day 4, the MN number between WT, SOD1^G93A^ treated and untreated conditions was comparable; however, from day 6 onwards, MNs co-cultured with untreated SOD1^G93A^ astrocytes displayed a constant viability decline [day 4: 100% viability; day 14: 4.9% viability]. Conversely, the number of MNs co-cultured with EV-treated SOD1^G93A^ astrocytes pre-treated with EVs was higher at each time point than MNs seeded on untreated astrocytes (Figure 1d).

We performed similar experiments using iAstrocytes in a phenotypic screen standardised with MN survival as the primary read-out for iAstrocyte toxicity [57]. We differentiated patient-derived iAstrocytes from induced neural progenitor cells (iNPCs) reprogrammed from skin fibroblasts of two healthy controls (CTR155, CTR3050), two C9orf72 patients (pat78, pat183), and two SOD1 patients (pat100, pat102) over our standardised 7-day differentiation protocol [56]. As to mouse astrocytes, iAstrocytes were pre-treated for 24h with EVs isolated from hMSCs stimulated with IFN-γ and co-cultured with murine Hb9-GFP+ motor neurons seeded on top (Figure 1e,f). Confirming our previous findings [30,56], iAstrocytes from C9orf72 and SOD1 patients were toxic to motor neurons (Figure 1g). Pre-treating iAstrocytes with EVs resulted in a significant rescue of MN survival after 3 days of co-culture in all patient donor cell lines. Of note, in the most toxic C9orf72 astrocyte line (pat183), astrocyte survival was 50% greater upon EV treatment.

Thus, exposure of mouse or patients’ ALS astrocytes to EVs significantly reduced their toxicity towards MNs.

### 3.2. MSC-Derived EVs Reduce the Pathological Activation and the Inflammatory Phenotype in Mouse SOD1^G93A^ Astrocytes

**Figure 2:**
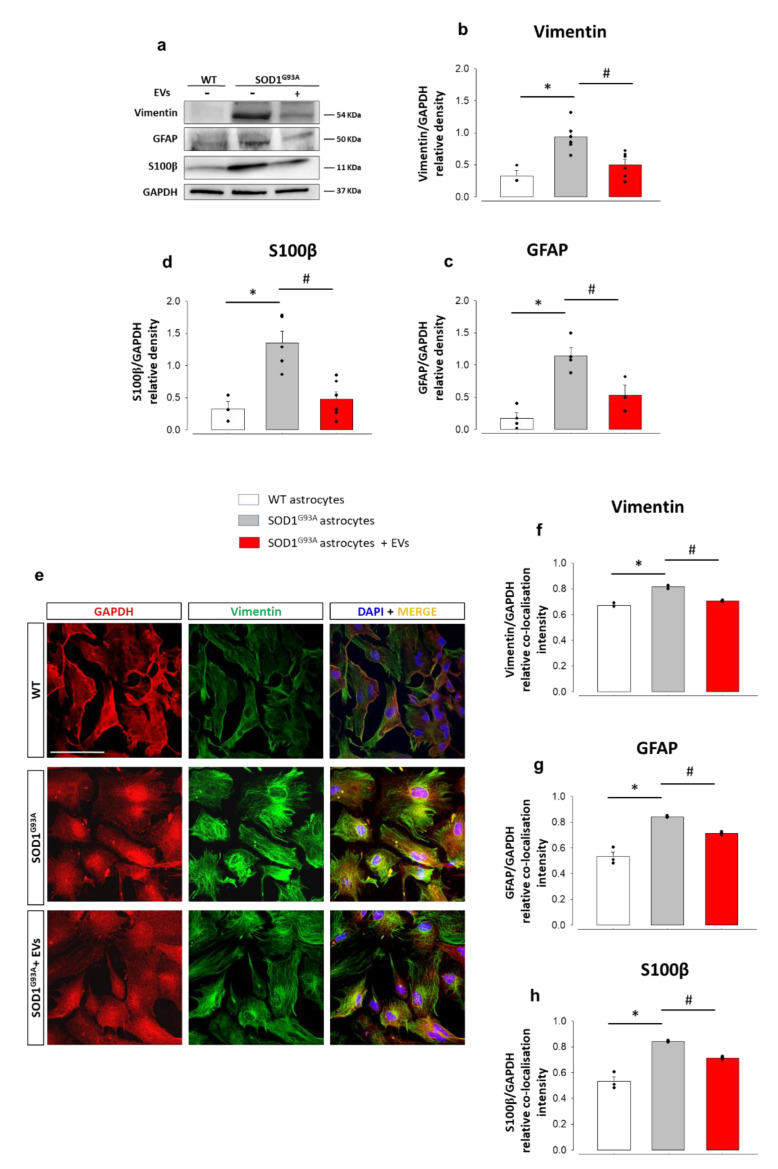
MSC-derived EVs reduce astrogliosis in astrocytes from the spinal cords of adult SOD1^G93A^mice. (**a**) Representative Western blots (WB) of immunoreactive bands for vimentin, GFAP, and S100β; (**b**–**d**) quantitative representation of WB densitometric expression signals for (**b**) vimentin, (**c**) GFAP, and (**d**) S100β normalized to glyceraldehyde 3-phosphate dehydrogenase (GAPDH), as housekeeping protein, in WT astrocytes, untreated SOD1^G93A^ astrocytes, and SOD1^G93A^ astrocytes treated for 24 h with MSC-derived EVs. Vimentin, GFAP, and S100β expression are significantly increased in untreated SOD1^G93A^ astrocytes vs. WT astrocytes (F_(2,12)_ = 10.998, *p* = 0.004; F_(2,8)_ = 17.903, *p* = 0.001; F_(2,13)_ = 9.653, *p* = 0.005, respectively), while the exposure to EVs significantly reduces their expression (F_(2,12)_ = 10.998, *p* = 0.01; F_(2,8)_ = 17,903, *p* = 0.02; F_(2,13)_ = 9.653, *p* = 0.01, respectively). Data are presented as means ± SEM of *n* = 3–6 independent experiments; statistical significance (* vs. WT and # vs. SOD1^G93A^ astrocytes) for *p* < 0.05 at least (one-way ANOVA, followed by Bonferroni post hoc test). (**e**) Representative confocal microscopy immunocytochemical images for GAPDH (red fluorescence), vimentin (green fluorescence), an’ 4′,6-diamidin-2-fenilindolo (DAPI, blue fluorescence) in WT astrocytes, SOD1^G93A^ astrocytes and EVs-treated SOD1^G93A^ astrocytes. Scale bar: 100 µm. (**f**–**h**) Quantitative representation of protein expression was calculated as the relative fluorescence intensity of the protein of interest co-localized with the stable reference protein GAPDH. The quantitative analyses of the relative co-localization intensity of fluorescence correlated with the protein expression level were performed by calculating the Manders and Costes co-localization coefficients [58,59], and normalizing the results with respect to the fluorescence intensity of the stable housekeeping protein GAPDH. (**f**) Vimentin, (**g**) GFAP and (**h**) S100β expression are significantly increased in untreated SOD1^G93A^ astrocytes vs. WT astrocytes [F_(2,6)_ = 98.542, *p* < 0.001; F_(2,6)_ = 47.483, *p* < 0.001; F_(2,6)_ = 61.283, *p* < 0.001, for vimentin, GFAP, and S100β, respectively], while the exposure to MSC-derived EVs significantly reduces their expression [F_(2,6)_ = 98.542, *p* < 0.001; F_(2,6)_ = 47.483, *p* < 0.001; F_(2,6)_ = 61.283, *p* < 0.02, for vimentin, GFAP and S100β, respectively]. Data are presented as means ± SEM of *n* = 3 independent experiments, run in triplicate; statistical significance for *p* < 0.05 at least (* vs. WT and # vs. SOD1^G93A^ astrocytes; one-way ANOVA, followed by Bonferroni post hoc test).

The involvement of astrocytes in propagating inflammation in ALS has been extensively described [31,61,62], and we have previously demonstrated that in vivo administration of MSCs reduced astrocyte pathological activation and IL-β and TNF-α expression in the spinal cord of SOD1^G93A^ mice [39]. Western blotting experiments, aimed at evaluating the effects of the exposure to MSC-derived EVs on astrocytes’ reactive phenotype markers, revealed that the expression of vimentin (Figure 2a,b), GFAP (Figure 2a,c), and S100β (Figure 2a,d) were significantly upregulated in SOD1^G93A^ astrocytes compared to WT astrocytes. Treating SOD1^G93A^ astrocytes with MSC-derived EVs reverted the up-regulation of vimentin, GFAP, and S100β to control levels. We confirmed the reduction of vimentin (Figure 2e,f), GFAP (Figure 2g and Figure S2a), and S100b (Figure 2h and Figure S2b) overexpression after exposure to MSC-derived EVs using semi-quantitative immunocytochemistry.

These findings demonstrate that EVs derived from IFNγ-stimulated MSCs efficiently reduce the expression of astrogliosis markers, thus reflecting a lower astrocyte pathological reactivity.

**Figure 3:**
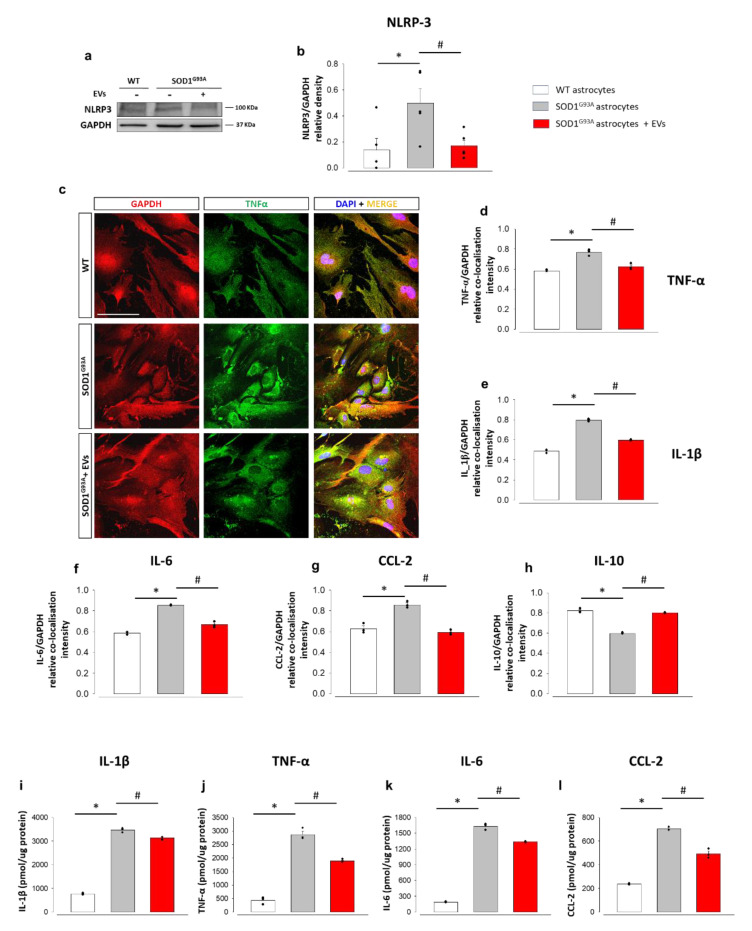
MSC-derived EVs reduce NLRP3-inflammasome, and the expression and release of pro-inflammatory cytokines, in mouse astrocytes. (**a**) Representative WB immunoreactive bands for NLRP3 in WT astrocytes, SOD1^G93A^ astrocytes and EV-treated SOD1^G93A^ astrocytes. (**b**) Quantitative representation of NLRP3 expression, normalized to GAPDH. Data are presented as means ± SEM of *n* = 5 independent experiments; statistical significance for *p* < 0.05 (* vs. WT and # vs. SOD1^G93A^ astrocytes; *p* = 0.008 vs. WT astrocytes; *p* = 0.02 vs. untreated SOD1^G93A^ astrocytes; F_(2,10)_ = 9.562; one-way ANOVA, followed by Bonferroni post hoc test). (**c**) Representative images for GAPDH (red fluorescence), TNF-α (green fluorescence), and DAPI (blue fluorescence), in WT astrocytes, SOD1^G93A^ astrocytes and EV-treated SOD1^G93A^ astrocytes. Scale bar: 100 µm. (**d**–**h**) Quantitative representation of fluorescence intensity (see legend to Figure 2 for details) for (**d**) TNF-α, (**e**) IL-1β, (**f**) IL-6, (**g**) CCL2, and (**h**) IL-10, in WT astrocytes, untreated SOD1^G93A^ astrocytes and EV-treated SOD1^G93A^ astrocytes. Data are presented as means ± SEM of *n* = 3 independent experiments, run in triplicate; statistical significance for *p* < 0.05 (* vs. WT and # vs. SOD1^G93A^ astrocytes; F_(2,6)_ = 40.686, *p* < 0.001; F_(2,6)_ = 443.910, *p* < 0.001; F_(2,6)_ = 174.847, *p* < 0.001; F_(2,6)_ = 50.196, *p* < 0.001; F_(2,6)_ = 256.549, *p* < 0.001 for TNF-α, IL-1β, IL-6, CCL2, and IL-10, respectively, in SOD1^G93A^ vs. WT; astrocytes; *p* = 0.002, *p* < 0.001, *p* < 0.001, *p* < 0.001 and *p* < 0.001 for TNF-α, IL-1β, IL-6, CCL2, and IL-10, respectively, in EV-treated SOD1^G93A^ vs. untreated SOD1^G93A^ astrocytes; one-way ANOVA, followed by Bonferroni post hoc test). (**i**–**l**) ELISA quantification of cytokines (pmol/ug of proteins), for (**i**) IL-1β, (**j**) TNF-α, (**k**) IL-6 and (**l**) CCL2, in the conditioned media of WT, untreated SOD1^G93A^ astrocytes, and EV-treated SOD1^G93A^ astrocytes. Data are presented as means ± SEM of *n* = 3 independent experiments, run in triplicate; statistical significance for *p* < 0.05 (* vs. WT and # vs. SOD1^G93A^ astrocytes; F_(2,6)_ = 202.041, *p* < 0.001;F_(2,6)_ = 1331.665, *p* < 0.001; F_(2,6)_ = 1314.021, *p* < 0.001; F_(2,6)_ = 269.710, *p* < 0.001, for TNF-α, IL-1β, IL-6 and CCL2, respectively, in SOD1^G93A^ vs. WT astrocytes; *p* < 0.001, *p* = 0.003, *p* < 0.001, *p* < 0.001, for TNF-α, IL-1β, IL-6, and CCL2, respectively, in EVs-treated SOD1^G93A^ vs. untreated SOD1^G93A^ astrocytes; one-way ANOVA, followed by Bonferroni post hoc test).

To investigate the possible impact of EV treatment on the astrocyte neuroinflammatory phenotype, we analysed the effects of MSC-derived EVs on the expression of the inflammasome complex NLRP3 and several pro- and anti-inflammatory factors. Western blots showed that NLRP3 expression increased in adult SOD1^G93A^ vs. age-matched WT astrocytes and was almost normalized after EV exposure (Figure 3a,b). We obtained the same results by semiquantitative confocal microscopy (Figure S3a,b). Confocal microscopy also showed that the expression of the pro-inflammatory cytokines TNF-α (Figure 3c,d), IL-1β (Figure 3e and Figure S4a), IL-6 (Figure 3f and Figure S4b), and CCL-2 (Figure 3g and Figure S4c) was increased in SOD1^G93A^ and brought to control levels in EV-treated SOD1^G93A^ astrocytes. In addition, the quantification of IL-1β, TNF-α, IL-6, and CCL-2 in WT and SOD1^G93A^ astrocyte culture medium by ELISA revealed a massive increase of these pro-inflammatory cytokines in SOD1^G93A^ astrocytes and a significant decrease after the treatment with MSC-derived EVs, which was more evident in the case of TNF-α and CCL2 (Figure 3i–l). Astrocytes also secrete anti-inflammatory cytokines that can ameliorate their activated phenotype [63]. We measured the expression of the anti-inflammatory cytokine IL-10 with confocal microscopy. IL-10 significantly decreased in adult mouse SOD1^G93A^ compared to WT astrocytes, but levels were restored after EV treatment (Figure 3h and Figure S4d).

**Figure 4:**
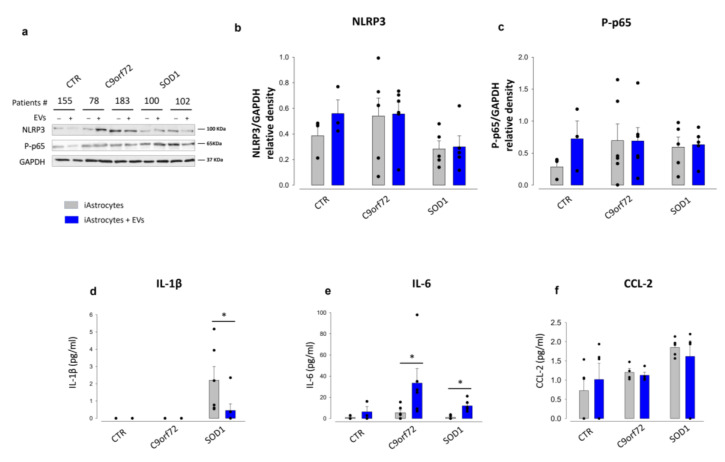
MSC-derived EVs do not affect the NLRP3-inflammasome, nor the p65 pathway in patients-derived iAstrocytes. (**a**). Representative WB immunoreactive bands for NLRP3 and P-p65 in patient-derived iAstrocytes differentiated from control (CTR) subjects, C9orf72, and SOD1 patients treated or not 24 h with human MSC-derived EVs. (**b**,**c**) Quantitative representation of WB densitometric expression signals for (**b**) NLRP3 and (**c**) P-p65 normalized to GAPDH. EV treatments did not show any significant effect on protein expression. Data are presented as means ± SEM of *n* = 3 independent experiments per patient. One control and 2 patients per group are included (One-way ANOVA, followed by Bonferroni post hoc test). (**d**–**f**) ELISA quantification of cytokines, reported as pg/mL, for (**d**) IL-1β, (**e**) IL-6 and (**f**) CCL2, in the conditioned media of iAstrocytes differentiated from control CTR, C9orf72, and SOD1 subjects treated or not 24 h with human MSC-derived EVs. Data are presented as means ± SEM of independent experiments as follows: *n* = 3–6 for one CTR, *n* = 4–6 for 2 C9 donors and *n* = 5–6 for 2 SOD1 donors; statistical significance for *p* < 0.05 at least (* *p* < 0.05 vs. untreated iAstrocytes; F_(2,30)_ = 8.936, F_(2,24)_ = 2.833, F_(2,30)_ = 2.677, for IL-1β, IL6 and CCL2, respectively, in untreated vs. EV-treated iAstrocytes; two-way ANOVA with Sidak’s multi comparison test).

We investigated in parallel the pro-inflammatory phenotype of patient-derived iAstrocytes. As opposed to mouse SOD1^G93A^ astrocytes, NLRP3 did not consistently increase in iAstrocytes from SOD1 and C9orf72 patients and, consequently, did not show significant changes after EV treatment (Figure 4a,b). We then assessed the levels of phospho-p65 (p-p65), the phosphorylated and active form of the nuclear factor kappa B (NFkB). We did not find any significant changes between p-p65 expression in iAstrocytes from healthy subjects vs. ALS patients, nor after EVs exposure (Figure 4a,c), Then, we assessed the levels of IL-1 β, IL-6, and CCL-2 secreted from human astrocytes. Samples from the healthy controls and C9orf72 patients showed levels of IL-1β below the detection limit. In contrast, and consistent with the murine astrocyte results, SOD1 iAstrocytes displayed higher IL-1β levels than controls, significantly reduced after EV treatment (Figure 4d). Secreted baseline levels of IL-6 (Figure 4e) were similar in patients and controls, while EVs exposure resulted in IL-6 increased expression. The baseline for CCL2 was higher in SOD1 and C9orf72 patients, as observed in the SOD1^G93A^ mouse astrocytes (Figure 4f), however, the exposure to MSC-derived EVs had no effect in patients or controls.

These data indicate that the inflammatory cytokine profiles of iAstrocytes and SOD1^G93A^ mouse astrocytes are clearly different and treatment with EVs resulted in a more marked decrease in neuro-inflammatory markers in mouse SOD1^G93A^ astrocytes than human SOD1 or C9orf72 iAstrocytes.

### 3.3. MSC-Derived EVs Modulate Nrf2 Nuclear Translocation and Promote Antioxidant Response in ALS Astrocytes

**Figure 5:**
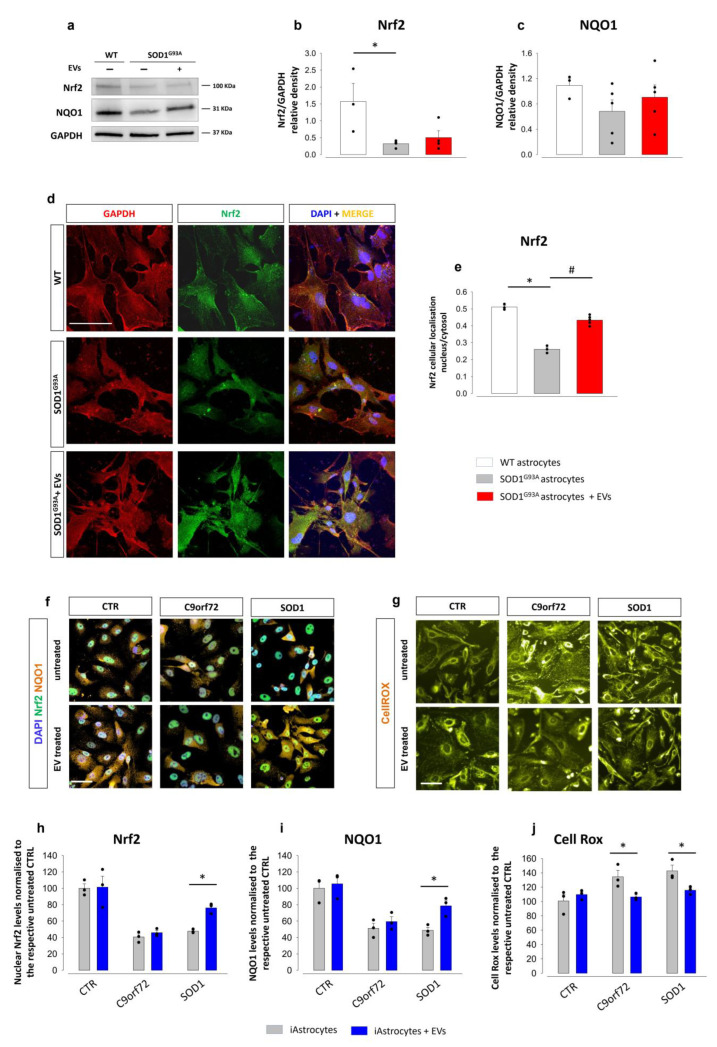
MSc-derived EVs positively modulate the Nrf2-NQO1 antioxidant pathway in mouse SOD1^G93A^ and patient-derived astrocytes. (**a**–**e**) Effect of MSC-derived EVs on oxidative stress in mouse SOD1^G93A^ astrocytes. (**a**) Representative WB images for Nrf2 and NQO1 in WT astrocytes, SOD1^G93A^ astrocytes and EV-treated SOD1^G93A^ astrocytes. (**b**,**c**) Quantitative analyses of WB experiments for Nrf2 and NQO1. Data are presented as means ± SEM of *n* = 3–4 (referred to Nrf2) and *n* = 3–5 (referred to NQO1) independent experiments, run in triplicate; statistical significance for *p* < 0.05 (* *p* < 0.05 vs. WT; Nrf2 F_(2,8)_ = 5.307; NQO1 F_(2,9)_ = 0.716; one-way ANOVA, followed by Bonferroni post hoc test). (**d**) Representative confocal microscopy images for GAPDH (red fluorescence), Nrf2 (green fluorescence) and DAPI (blue fluorescence), in WT, SOD1^G93A^ astrocytes and EV-treated SOD1^G93A^ astrocytes. Scale bar: 100 µm. (**e**) Quantitative representation of fluorescence intensity related to the nucleus/cytosol cellular localization ratio. Data are presented as means ± SEM of *n* = 3–5 independent experiments, run in triplicate; statistical significance for *p* < 0.05 (* *p* < 0.001 vs. WT; # *p*< 0.001 vs. untreated SOD1^G93A^ astrocytes; F_(2,6)_ = 307.883; one-way ANOVA, followed by Bonferroni post hoc test). (**f**–**j**) Effect of MSC-derived EVs in human iAstrocytes. (**f**) Representative immunofluorescence images of Nrf2 (green fluorescence) and NQO1 (orange fluorescence) in untreated iAstrocytes and iAstrocytes exposed to human MSC-derived EVs. Scale bar: 100 µm. (**g**) Representative immunofluorescence images of reactive oxygen species stained with CellROX*^®^* probe (orange fluorescence) in controls (CTR), untreated iAstrocytes and iAstrocytes exposed to human MSC-derived EVs. Scale bar: 100 µm. (**h**) Quantification of fluorescence for total Nrf2 expression in controls (CTR), C9orf72, and SOD1 patients, treated or not with human MSC-derived EVs. (**i**) Quantification of fluorescence for total NQO1 expression. Data are expressed as means ± SEM of *n* = 3 independent experiments. Statistical significance for *p* < 0.05 (* *p* < 0.05 vs. untreated iAstrocytes; F_(2,17)_ = 42.335 for Nrf2; F_(2,17)_ = 26.163 for NQO1; two-way ANOVA with Sidak’s multi comparison test). (**j**) Quantitative analyses of reactive oxygen species stained with CellROX*^®^* probe, in controls (CTR), C9orf72, and SOD1 patients treated or not with EVs. Data are expressed as means ± SEM, *n* = 3 independent experiments, including two technical replicates per experiment. Statistical significance for *p* < 0.05 (* *p* < 0.05 vs. untreated iAstrocytes; F_(2,17)_ = 6.605; two-way ANOVA with Sidak’s multi comparison test).

Activation of the Nrf2 antioxidant pathway ameliorates astrocyte toxicity [64]. We thus investigated whether Nrf2 and downstream NQO1 activation is a mechanism of EV-mediated reduction of astrocyte toxicity towards MNs. Western blot experiments showed that the total expression of Nrf2 was markedly decreased in SOD1^G93A^ compared to age-matched WT astrocytes, confirming a deficit of the antioxidant response, and that exposure of SOD1^G93A^ astrocytes to mouse MSC-derived EVs did not affect Nrf2 expression (Figure 5a,b). NQO1 expression was slightly reduced in SOD1^G93A^ astrocytes, and EV treatment did not affect the expression of NQO1 (Figure 5a,c). Since Nrf2 exerts its activity in the nucleus, where it promotes the transcription of cytoprotective enzymes, we assessed the translocation of Nrf2 into the nucleus by confocal microscopy, analysing the nucleus/cytoplasm cellular localization ratio of the Nrf2 factor (Figure 5d). The nuclear localization of Nrf2 in SOD1^G93A^ astrocytes was significantly lower than in WT astrocytes. Treatment with MSC-derived EVs led to almost complete restoration of Nrf2 levels in the nuclear compartment (Figure 5e). Thus, even if total Nrf2 did not change following EV exposure, there was an augmentation of Nrf2 nuclear translocation, which we predict boosts the antioxidant response in SOD1^G93A^ astrocytes.

To assess whether MSC-derived Evs also improved the antioxidant response in patient-derived iAstrocytes, we quantified the level of Nrf2 and NQO1 (via immunofluorescence) and reactive oxygen species (ROS) using the CellROX cell dye (see representative Figure 5f,g) in control, C9orf72, and SOD1 iAstrocytes treated or not with EVs. Both nuclear Nrf2 and total NQO1 levels were significantly lower in untreated iAstrocytes than in controls (Figure 5h,i). Treating iAstrocytes with EVs, however, led to an increase in nuclear Nrf2 and total NQO1 levels in SOD1 iAstrocytes. To explore whether the increase in the antioxidant response led to a consequent reduction of reactive oxygen species, we performed a CellROX*^®^* live cell assay. iAstrocytes displayed higher levels of intracellular reactive oxygen species (ROS) in C9orf72 and SOD1 patients compared to controls; interestingly, ROS levels were significantly reduced upon EV treatment in both groups (Figure 5j).

The combination of increased activation of Nrf2/NQO1 signalling with a reduction in ROS production indicates that promoting the antioxidant response is one of the mechanisms involved in MSC-derived EV-mediated neuroprotection in human astrocytes.

### 3.4. EV-Shuttled miRNAs Are Responsible for the EV-Mediated Neuroprotection

**Figure 6:**
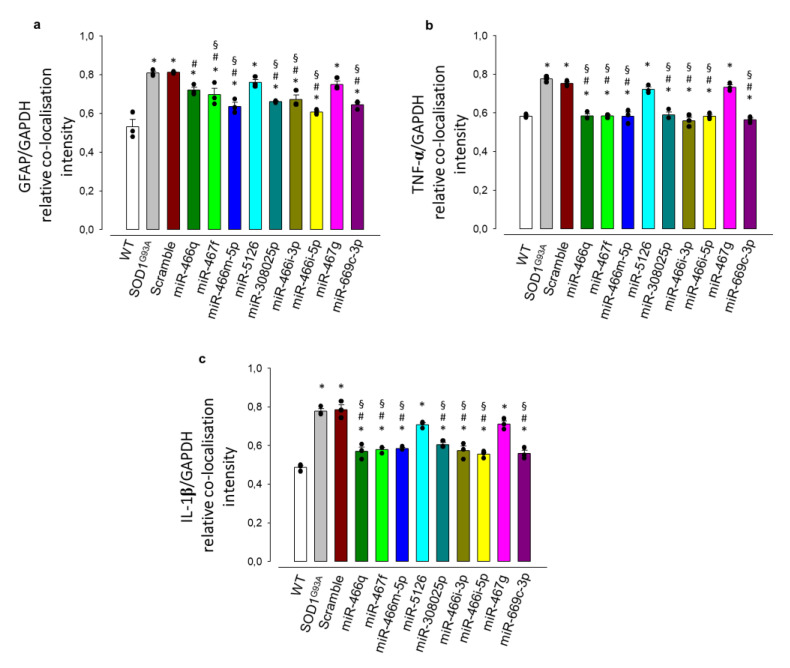
miRNAs shuttled by EVs mediate the reduction of astrogliosis and inflammatory cytokines expression in SOD1^G93A^astrocytes. (**a**–**c**) Quantitative representation of protein expression as per relative fluorescence intensity with respect to the stable housekeeping protein GAPDH. The quantitative analyses of the relative co-localization intensity of fluorescence correlated with the protein expression level were performed as described in the legend to Figure 2. (**a**) GFAP, (**b**) TNF-α and (**c**) IL-1β expression are significantly increased in untreated or scramble-miRNA-treated SOD1^G93A^ astrocytes vs. WT astrocytes. The exposure to seven out of nine specific synthetic mimics of miRNAs shuttled by EVs significantly reduces the expression of the GFAP, TNF-α, and IL-1β, indicating a decreased astrocyte reactivity. Data are presented as means ± SEM of *n* = 3 independent experiments, run in triplicate; statistical significance for *p* < 0.05 at least (* *p* < 0.001 vs. WT astrocytes; # *p* < 0.001 vs. SOD1^G93A^ astrocytes; § *p* < 0.001 vs. SOD1^G93A^ astrocytes + scrambled miRNA; F_(11,24)_ = 17.967, F_(11,24)_ = 49.372, F_(11,24)_ = 38.634, for GFAP, TNF-α, and IL-1β, respectively; one-way ANOVA, followed by Bonferroni post hoc test).

We recently found that miRNA expression significantly increased in IFNγ-primed MSCs [45]. The same overexpressed miRNAs were present in EVs isolated from MSCs, which proved to affect the neuroinflammatory phenotype of activated mouse microglia [45]. As in the previous study, we transfected SOD1^G93A^ astrocytes with the EV-shuttled miRNA synthetic mimics. After 48 h of transfection with single mimics, we observed that seven out of nine miRNA mimics induced a significant reduction of GFAP, TNF-α, and IL-1β expression, except for miR-5126 and miR-467g (Figure 6a–c; Supplementary Table S4).

**Figure 7:**
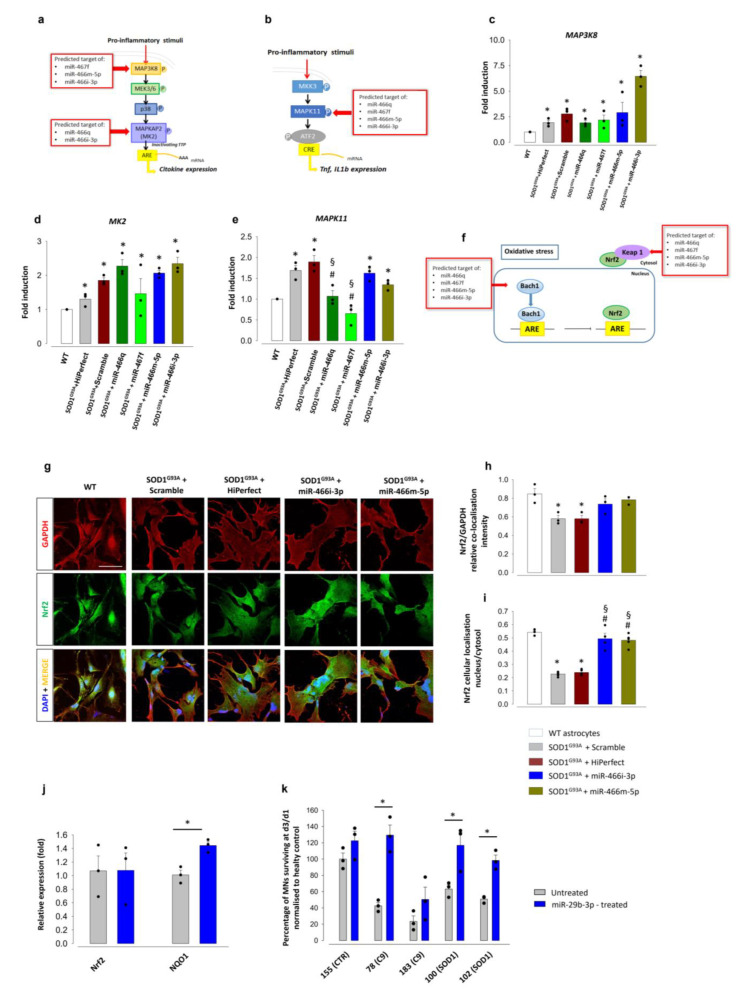
EV-shuttled miRNAs modulate the pathological reactive phenotype, Nrf2 expression, and neurotoxicity in SOD1^G93A^ astrocytes and iAstrocytes. Graphical representation of (**a**) MAP3K8/MK2, (**b**) MAPK11 and (**f**) Nrf2 pathways. RT-qPCR of (**c**) Map3k8, (**d**) Mapkapk2, and (**e**) Mapk11 expression in WT, SOD1^G93A^ astrocytes + Hiperfect, SOD1^G93A^ astrocytes + scrambled miRNAs, and SOD1^G93A^ astrocytes + miRNAs. Data are presented as means ± SEM of *n* = 3 independent experiments, run in triplicate; statistical significance for *p* < 0.05 (* *p* < 0.05, vs. WT; # *p* < 0.01 vs. SOD1^G93A^ astrocytes + Hiperfect; § *p* < 0.01 vs. SOD1^G93A^ astrocytes + scrambled miRNA; F_(6,14)_ = 11.738, F_(6,14)_ = 6.321 and F_(6,14)_ = 14.786 for Map3k8, Mapkapk2 and Mapk11, respectively; one-way ANOVA followed by Bonferroni post hoc test). (**g**) Representative images for GAPDH (red fluorescence), Nrf2 (green fluorescence) and DAPI (blue fluorescence), in WT, SOD1^G93A^ astrocytes, and SOD1^G93A^ astrocytes treated with miRNAs. Scale bar: 100 µm. (**h**,**i**) Quantitative representation of fluorescence intensity (see Figure 2) for (**h**) Nrf2 total expression and (**i**) Nrf2 cellular localization expressed as nuclear/cytoplasm ratio. Data are presented as means ± SEM of *n* = 3 independent experiments (referred to Nrf2 total expression) and *n* = 3–6 independent experiments (referred to Nrf2 cellular localization); statistical significance for *p* < 0.05 (* *p* < 0.001 vs. WT; # *p* < 0.001 vs. SOD1^G93A^ astrocytes + HighPerfect; § *p* < 0.001 vs. SOD1^G93A^ astrocytes + scrambled miRNA; Nrf2/GAPDH, F_(4,10)_ = 7.507; Nrf2 nucleus/cytoplasm, F_(4,10)_ = 214,640; two-way ANOVA followed by Bonferroni post hoc test). (**j**) RT-qPCR validation of Nrf2 and NQO1 expression in control iAstrocytes after miR-29b3P transfection. Data are presented as means ± SEM of *n* = 3 independent experiments; statistical significance for * *p* < 0.05 vs. control iAstrocytes (NQO1, t = −4.882; Nrf2, t = −0.0197; two-tailed Student’s *t*-test). (**k**) Quantification of MN survival expressed as the percentage of MNs with axon (calculated respect to the total number of MNs at day 1) after 3 days of co-culture with untreated iAstrocytes or iAstrocytes transfected with miRNA mimic miR-29b3P. Data are presented as means ± SEM of *n* = 3 biological replicates; statistical significance * *p* < 0.05 vs. untreated iAstrocytes; two-tailed Student’s *t*-test.

We subsequently assessed the anti-inflammatory effect of four miRNAs (miR-466q, miR-467f, miR-466m-5p, miR466i-3p) which were found upregulated both in IFNγ-primed-MSCs and in their derived EVs [45]. According to our recent publication, and using the miRWalk and KEGG & Panther Classification System databases [45], we focused on the effect of the four mimics on components of the p38 MAPK signalling pathway that is involved in the production of pro-inflammatory cytokines [65], including Map3k8 (target of miR67f, 466i-3p, 466m-5p), Mk2 (target of miR-466q and miR-466i-3p) (Figure 7a), and Mapk11, which promotes TNF-α and IL-1β synthesis through phosphorylation of the downstream activating transcription factor-2 (ATF-2) [66,67] (Figure 7b). RT-PCR analysis showed that none of the four mimics reverted the increased expression of Mapk8 and Mk2 observed in SOD1^G93A^ astrocytes (Figure 7c,d), while mimics of miR-466q and miR-467f normalised the overexpression of Mapk11 to WT levels, (Figure 7e). These results suggest a selective anti-inflammatory potential of miR-466q and miR-467f in SOD1^G93A^ astrocytes.

Additionally, we investigated the ability of miR-466m-5p and miR-466i-3p to modulate the antioxidant pathway linked to Nrf2, considering that the transcription factors BTB and CNC homology 1 (Bach1) and Kelch-like ECH associated protein 1 (Keap1) are two endogenous inhibitors of Nrf2 and predicted targets of miR-466i-3p and miR-466m-5p. Bach1 competes for the same binding site as Nrf2 on the enhancer antioxidant response element (ARE), preventing its activation and the synthesis of antioxidant enzymes [68]. Keap1 binds Nrf2 in the cytoplasm and prevents its translocation into the nucleus [69] (Figure 7f). Confocal microscopy experiments showed that the total expression of Nrf2, which decreased in SOD1^G93A^ astrocytes compared to WT astrocytes, was not significantly affected by transfection with either mimic (Figure 7g,h). As also shown above, the nuclear expression of Nrf2 decreased in SOD1^G93A^ astrocytes vs. WT astrocytes. Interestingly, the treatment with miR-466m-5p or miR-466i-3p reversed the defective translocation of Nrf2 into the nuclear compartment (Figure 7g,i).

These results suggest a possible mechanism for the MSC-derived EV-induced nuclear translocation of Nrf2 observed in SOD1^G93A^ astrocytes.

Similarly to mouse MSCs [45], we performed microarray analysis of a miRNAs expressed by hMSCs (Supplementary Table S5) and found that miR-29b3P was one of the upregulated miRNAs in IFN-γ-primed hMSCs. Interestingly, this miRNA acts as a direct target of Nrf2 [70] and regulates the Nrf2 axis by silencing the downstream inhibitor Bach2 (www.mirdb.org; http://mirdb.org/cgi-bin/target_detail.cgi?target; accessed on 14 March 2022; ID = 2387339; [71,72]). Indeed, the expression of miR-29b-3p reduced ROS production in cancer cells [73]. We assessed whether this miRNA could be responsible for the antioxidant response triggered in iAstrocytes by MSC-derived EVs and, consequently, for MN rescue. We transfected iAstrocytes with miR-29b-3p mimic before seeding motor neurons. Because miR-29b-3p has been linked to activation of the antioxidant pathway via Nrf2 [70], we performed RT-qPCR to investigate the expression of the genes encoding for Nrf2 and NQO1 in control iAstrocytes transfected with the miR-29b-3p mimic (Figure 7j). Consistent with the Nrf2 downstream action of miR-29b-3p, miR-29b-3p transfection had no effect on Nrf2, but increased NQO1 expression. Similarly to EVs, miR-29b-3p mimic restored MN survival in co-culture in three out of four lines, leading to a 40% increase viability in the most aggressive ALS C9orf72 line (Figure 7k).

These data suggest that miR-29b-3p may mediate MN rescue through antioxidant effects.

## 4. Discussion

We show that in vitro exposure of ALS astrocytes to MSC-derived EVs in human and mouse models reduced their neurotoxicity towards MNs, possibly by EV-shuttled miRNAs. Our results support previous findings showing that MSC-conditioned medium exerts a comparable ameliorative effect in different in vitro models of ALS. To our knowledge, we describe here the first demonstration that MSC-derived EVs are directly responsible for reducing astrocyte neurotoxicity.

We studied the effects of human MSC-derived EVs on i-Astrocytes from two SOD1 and two C9*orf*72 ALS patients, and two healthy controls, unveiling the amelioration of the i-Astrocyte phenotype and the reduction of neuronal toxicity in specific ALS genetic subtypes. Consistently, pre-treating adult SOD1^G93A^ mouse astrocytes with mouse MSC-derived EVs also reduced neurotoxic effects. Thus, our evidence gathered on iAstrocytes and SOD1^G93A^ mouse astrocytes demonstrated that both in vitro models are useful to shed light on the mechanisms of MSC-derived EV effects.

In the mouse cell model, exposure to the MSC-derived EVs reduced the expression of the astrogliosis markers vimentin, GFAP, and S100β, NLRP3 inflammasome, and the expression and secretion of IL-1β, TNF-α, IL-6, and CCL2 pro-inflammatory cytokines. In contrast, they did not increase antioxidants via Nrf2 or NQO1. These results suggest that in SOD1^G93A^ astrocytes, MN rescue is primarily based on reduced inflammatory mechanisms, while in the i-Astrocyte the modulation of inflammation might play a minor role, with EVs being more effective in modulating the oxidative stress. However, consistent with the mouse counterpart, iAstrocytes displayed high levels of IL-1β and CCL2, and EV treatment significantly decreased IL-1β, but not CCL2.

The differences between the anti-inflammatory or antioxidant effects of EVs in adult mouse astrocytes and iAstrocytes can rely on their different origins. Adult mouse astrocytes from the spinal cord of symptomatic SOD1^G93A^ mice mature in vivo in a neuroinflammatory pathological environment until isolation [53]. In human iAstrocytes, we found a mild increase of neuroinflammatory markers, but instead a higher ROS accumulation and a lower total Nrf2, the master regulator of the antioxidant cascade, and EV treatment successfully overturned these pathological features. Moreover, SOD1 and C9orf72 astrocytes differ in their pro-inflammatory profile, with the expression of IL-1β being higher in iAstrocytes from SOD1 patients, and this aspect is worth further investigation.

We have profiled the miRNAs in mouse MSC-derived EVs and used their synthetic miRNA mimics to assess the effect on the reactive astrocyte properties. We found that seven out of nine mimics of miRNAs upregulated in IFN-γ-primed MSCs reduced the pathological reactive astrocyte phenotype. Four of them, miR-466q, miR-467f, miR-466m-5p, and miR-466i-3p, overexpressed in both MSCs and EVs, were tested for their ability to modulate the inflammatory response. After in silico analysis aimed at identifying specific inflammatory pathways, we focused on the NFҡB and MAPK pathways. Transfection with each single miRNA mimic had a modest effect on the p38 MAPK pathway; in contrast, *MapK11*, another predicted target of the four miRNAs, which promotes TNF-α and IL-1β synthesis, was significantly upregulated in SOD1^G93A^ astrocytes and reduced by miR-466q or miR-467f. The identified miRNAs also acted on oxidative stress. Indeed, in silico analysis indicated that miR-466i-3p and miR-466m-5p are potential modulators of Keap1 and Bach1, two endogenous inhibitors of Nrf2 that prevent its nuclear translocation and activity [68,69]. Interestingly, miR-466m-5p promoted Nrf2 translocation, possibly boosting the antioxidant response. Thus, MSC-derived miRNAs could be responsible for the beneficial effect of the EVs in mouse astrocytes by modulating both the inflammatory and the antioxidant pathways.

In human iAstrocytes, the addition of the synthetic miR-29b-3p mimic to C9*orf*72 and SOD1 iAstrocytes before co-culture with MNs significantly upregulated NQO1 and rescued MN survival. Further work is required to elucidate the pathways regulated by the EV-shuttled miRNAs.

We have shown for the first time that the application of MSC-derived EVs to SOD1 and C9*orf*72 patients’ astrocytes and adult mouse SOD1^G93A^ astrocytes significantly rescued MN death, suggesting the possibility of replacing the MSC cell grafts in ALS with a less invasive and immunogenic administration of EVs [42,74]. Accordingly, the literature reports that EVs from adipose stem cells ameliorate the disease course in SOD1^G93A^ mice [75]. EVs have been demonstrated to play a key role in pathological processes since their cargo can contribute to the spread or modulation of a disease, but indeed they can be also exploited as a therapeutic strategy to overcome neurodegeneration associated with CNS pathologies, and this has been demonstrated with particular emphasis on Alzheimer’s disease and Parkinson’s disease, other than amyotrophic lateral sclerosis [76,77]. In particular, EVs secreted by MSCs have aroused considerable interest as a potential cell-free therapy [78,79]. In the last decade, it has become evident to the scientific community that EV cargo can indeed contain molecules such as miRNAs, as we confirmed in the former and present paper, that can be themselves new targets or can target key cellular pathways in the receiving cells, suggesting that they could be considered as a tool for new potential gene therapies. Moreover, EVs can easily cross the blood–brain barrier and can be exploited to deliver drugs [80,81].

In this framework, our findings surely support a new potential approach further contributing to the landscape of innovative ALS therapies. Very recently, this scenario has been strongly enriched with the ground-breaking discovery and application of the antisense oligonucleotide (ASOs) Tofersen, which efficiently mediates the degradation of superoxide dismutase 1 (SOD1) messenger RNA to reduce SOD1 protein synthesis in human patients affected by inherited SOD1 mutations [82,83]. At the same time, Mueller et al. showed the efficacy of a single infusion of adeno-associated virus encoding a microRNA targeting the SOD1 [84]. These results are also supported by the possibility of exploiting the CRISPR base editors in SOD1^G93A^ mice, reinforcing the potentiality of gene therapies [85]. Of note, all the above experimental treatments are delivered by an invasive route, such as intrathecal infusion, and are limited to SOD1 or C9ORF72 mutations. Conversely, EVs can be administered by a non-invasive route, such as intranasally, entering the brain quickly and efficiently, thus representing a very translational opportunity for the treatment of neurological diseases [86,87,88].

Based on the above evidence, as the subsequent translational application of the data reported in this paper, our research groups are currently involved in carrying out studies aimed at testing the efficacy of MSC-derived EVs intranasally administered in SOD1^G93A^ ALS mice by performing a comprehensive panel of in vivo functional studies, as well as ex vivo histological and molecular analyses, as previously reported [89,90,91,92].

Our results pave the way also for using miRNA-loaded EV-mimicking synthetic particles, bypassing the culture and amplifying MSCs. Moreover, they are significant because they extend beyond SOD1 as a model for ALS and demonstrate a broader therapeutic potential for EVs in different subtypes of ALS.

## Data Availability

Raw data were generated at the University of Genoa and at the University of Sheffield. The experimental datasets generated and/or analysed during the current study are available from the corresponding authors upon reasonable request.

## Supplementary Materials

The following supporting information can be downloaded at: https://www.mdpi.com/article/10.3390/cells11233923/s1. Figure S1: Confocal microscopy representative images showing MN/astrocyte co-cultures labelled with specific markers for astrocytes and motor neurons. Figure S2: Immunofluorescence representative images showing iAstrocytes and MN/iAstrocyte co-cultures labelled with specific markers for astrocytes and motor neurons. Figure S3: Characterization of exocytosis markers and size of the extracellular vesicle populations in IFN𝛾-stimulated MSCs. Figure S4: MSC-derived EVs reduce astrogliosis in astrocytes from the spinal cord of adult SOD1^G93A^ mice. Figure S5: MSC-derived EVs reduce the NLRP3 inflammasome in astrocytes from the spinal cord of adult SOD1^G93A^ mice. Figure S6: MSC-derived EVs reduce the expression of pro-inflammatory cytokines in astrocytes of adult SOD1^G93A^ mice. Table S1: Details of fibroblast donors. Table S2: List and sequence of the synthetic mimics used for mouse and human astrocyte transfections. Table S3: Details of antibodies. Table S4: Percentage of variation of GFAP, TNF-α and IL-1β expression in SOD1^G93A^ mouse-derived astrocytes after 48h transfection with single miRNA synthetic mimics vs. untreated SOD1^G93A^ astrocytes. Table S5: List of the up-regulated miRNAs detected in human MSCs (hMSCs) primed with IFN-γ.
