# Supplementary material for: Micro-RNAs Shuttled by Extracellular Vesicles Secreted from Mesenchymal Stem Cells Dampen Astrocyte Pathological Activation and Support Neuroprotection in In-Vitro Models of ALS
Source: Cells. 2022 Dec 4;11(23):3923. doi: 10.3390/cells11233923 (PMC9741322; doi:10.3390/cells11233923)
Supplement: Supplementary file 1 [file cells-11-03923-s001.zip › cells-1934958-SM.pdf]

cells MDPI

Supplementary materials

Micro-RNAs Shuttled by Extracellular Vesicles Secreted from Mesenchymal Stem Cells Dampen Astrocyte Pathological Activation and Support Neuroprotection in in-vitro Models of ALS

Francesca Provenzano [1,†], Sophie Nyberg [2,†], Debora Giunti [3,4], Carola Torazza [1], Benedetta Parodi [3], Tiziana Bonifacino [1,5], Cesare Usai [6], Nicole Kerlero de Rosbo [4,7], Marco Milanese [1,4,*], Antonio Uccelli [3,4], Pamela Jean Shaw [2], Laura Ferraiuolo [2,*,‡] and Giambattista Bonanno [1,4,‡]

[1] Department of Pharmacy (DIFAR), University of Genoa, Viale Cembrano 4, 16148 Genova, Italy; francescaprovenzano.fp@gmail.com (F.P.); torazza@difar.unige.it (C.T.); tiziana.bonifacino@unige.it (T.B.); giambattista.bonanno@unige.it (G.B.)
[2] Sheffield Institute for Translational Neuroscience (SITraN), University of Sheffield, 385A Glossop Road, Sheffield S10 2HQ, UK; sophie.nyberg@mndassociation.org (S.N.); pamela.shaw@sheffield.ac.uk (P.J.S.)
[3] Department of Neurosciences, Rehabilitation, Ophthalmology, Genetics, Maternal and Child Health (DINOGMI), University of Genoa, Largo Paolo Daneo, 316132 Genoa, Italy; dgiunti@neurologia.unige.it (D.G.); benedetta.parod@gmail.com (B.P.); auccelli@neurologia.unige.it (A.U.)
[4] IRCCS Ospedale Policlinico San Martino, Largo Rosanna Benzi 10, 16132 Genoa, Italy; nkder.work@gmail.com
[5] Inter-University Center for the Promotion of the 3Rs Principles in Teaching & Research (Centro 3R),Pisa, 56122, Italy
[6] Institute of Biophysics, National Research Council (CNR), Via De Marini 6, 16149 Genoa, Italy; cesare.usai@ibf.cnr.it
[7] TomaLab, Institute of Nanotechnology, National Research Council (CNR), Piazzale Aldo Moro 5, 0018 Rome, Italy
* Correspondence: marco.milanese@unige.it (M.M.); l.ferraiuolo@sheffield.ac.uk (L.F.); Tel.: +39-01-0335-2046 (M.M.); +44-(0)114-222-2257 (L.F.)
† These authors contributed equally to this work.
‡ Joint senior authors.

Supplementary materials & legends

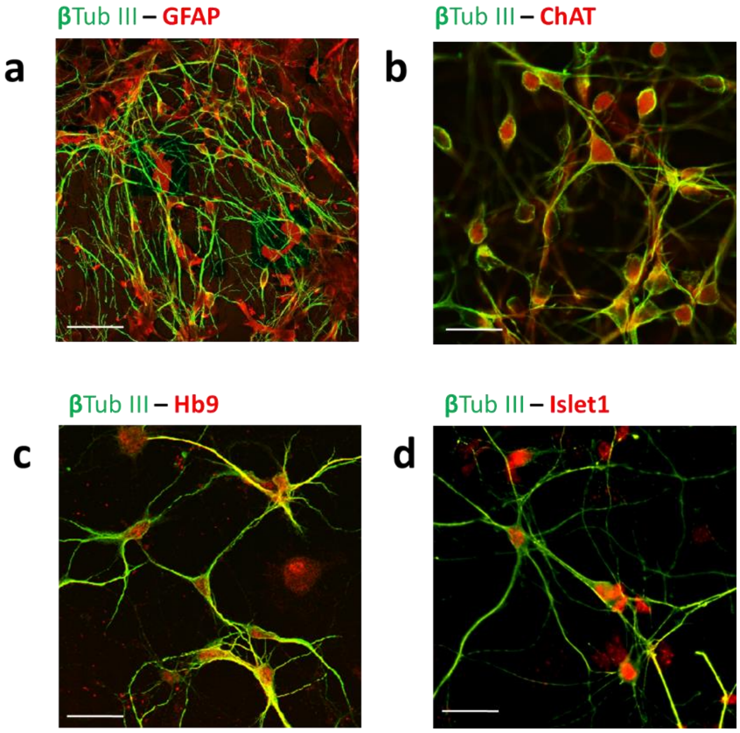

Supplementary figure 1. Confocal microscopy representative images showing MN/astrocyte co-cultures labelled with specific markers for astrocytes and motor neurons.
Representative confocal microscopy immunocytochemical images for beta-tubulin-III (βTub-III, green fluorescence) and GFAP (red fluorescence) (**a**), βTub-III (green fluorescence) and choline acetyltransferase (ChAT, red fluorescence) (**b**), βTub-III (green fluorescence) and homeobox protein HB9 (HB9, red fluorescence) (**c**) and, βTub-III (green fluorescence) and homeobox protein Islet1 (Islet1, red fluorescence) (**d**). Scale bar: 100 μm for panel a and 50 μm for panels b-d.

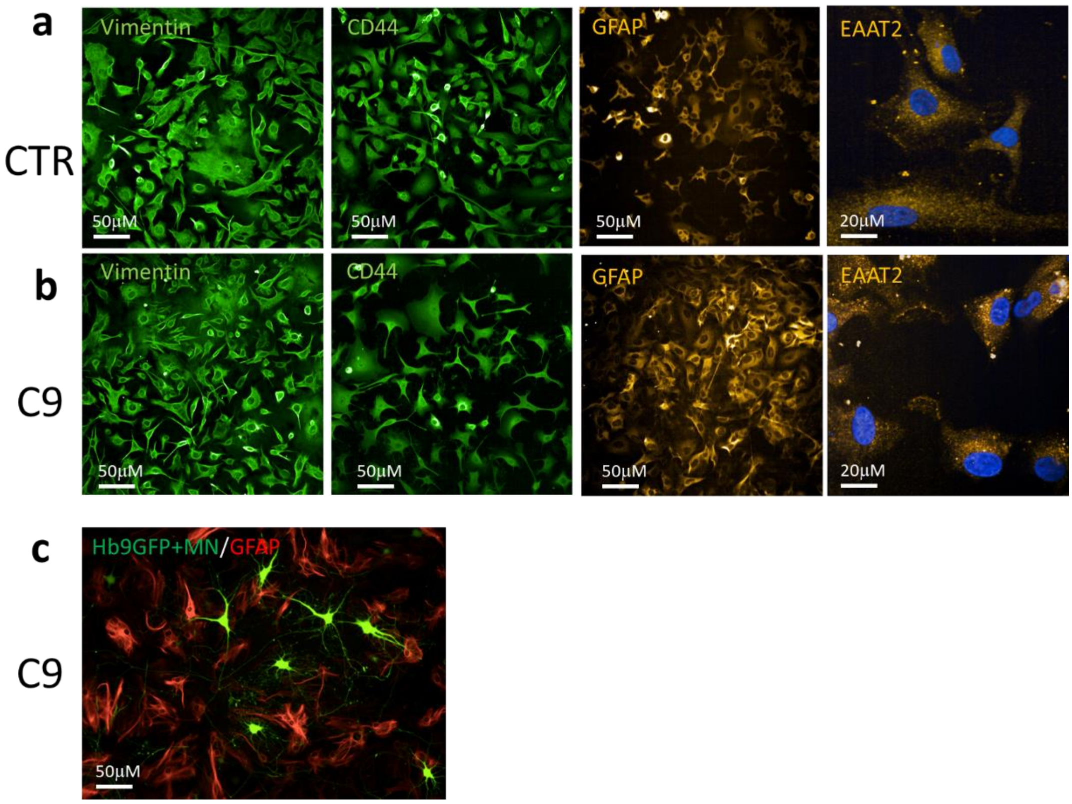

Supplementary figure 2. Immunofluorescence representative images showing iAstrocytes and MN/iAstrocyte co-cultures labelled with specific markers for astrocytes and motor neurons.

Representative images of iAstrocytes differentiated from induced neural progenitor cells (iNPCs), reprogrammed from fibroblast of healthy donors (CTR, panel a) and ALS patients (C9, panel b), stained for cell identity markers Vimentin, CD44, GFAP and EAAT2.

Panel (c) shows a representative image of co-cultures with Hb9 GFP+ mouse motor neurons (green native fluorescence) and human astrocytes from ALS donors (C9) stained with GFAP (red fluorescence).

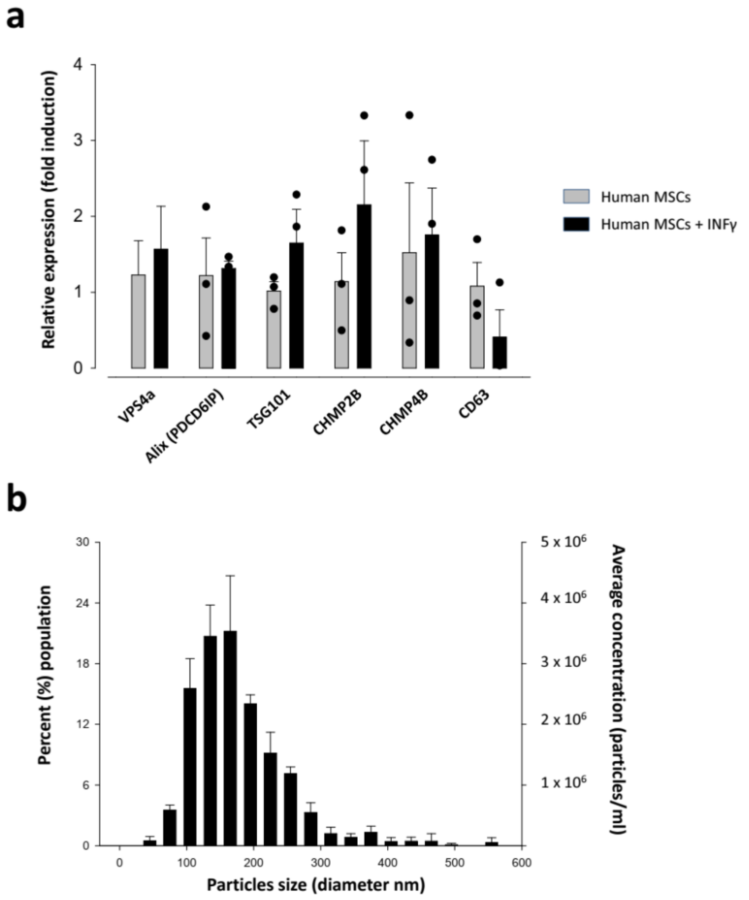

Supplementary figure 3. Characterization of exocytosis markers and size of the extracellular vesicle populations in IFNγ-stimulated MSCs.

(**a**) RT-qPCR quantification showing relative gene expression of exocytosis markers VPS4A, ALIX, TSG101, CHMP2B, CHMP4B and CD63 in human MSCs untreated or stimulated with IFNγ. Gene expression was normalised to GAPDH and reported as a fold induction in stimulated vs. untreated cells. IFNγ -stimulation did not show any significant effect on gene expression. Data are presented as means ± SEM of 3 independent experiments (One-way ANOVA, followed by Bonferroni post hoc test). (**b**) Size distribution of extracellular vesicles isolated from IFNγ-stimulated human MSCs as assessed by Zetaview, with size brackets reported as a percentage of the total population (left y axis) and average particle concentration/ml (right y axis). Data are expressed as means ± SEM, of 3 independent experiments.

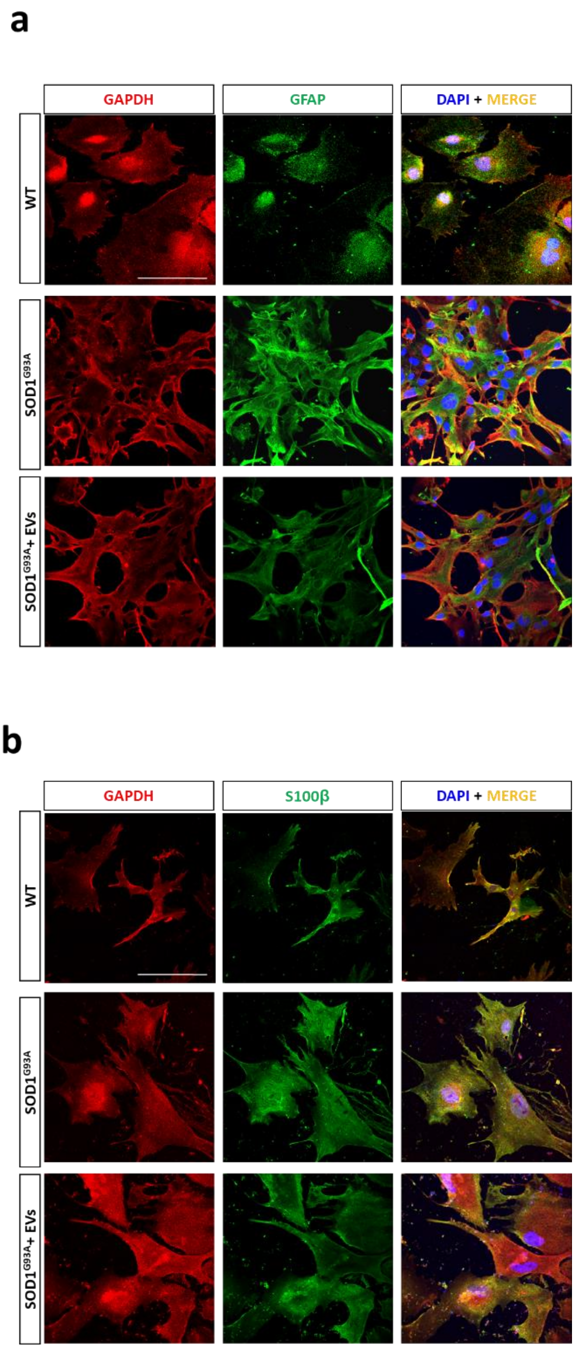

Supplementary figure 4. MSC-derived EVs reduce astrogliosis in astrocytes from the spinal cord of adult SOD1G93A mice.
Representative confocal microscopy immunocytochemical images for GAPDH (red fluorescence), (**a**) GFAP, and (**b**) S100β (green fluorescence), and 4',6-diamidin-2-fenilindolo (DAPI, blue fluorescence) in WT astrocytes, SOD1G93A astrocytes and EVs-treated SOD1G93A astrocytes. Scale bar: 100 μm.

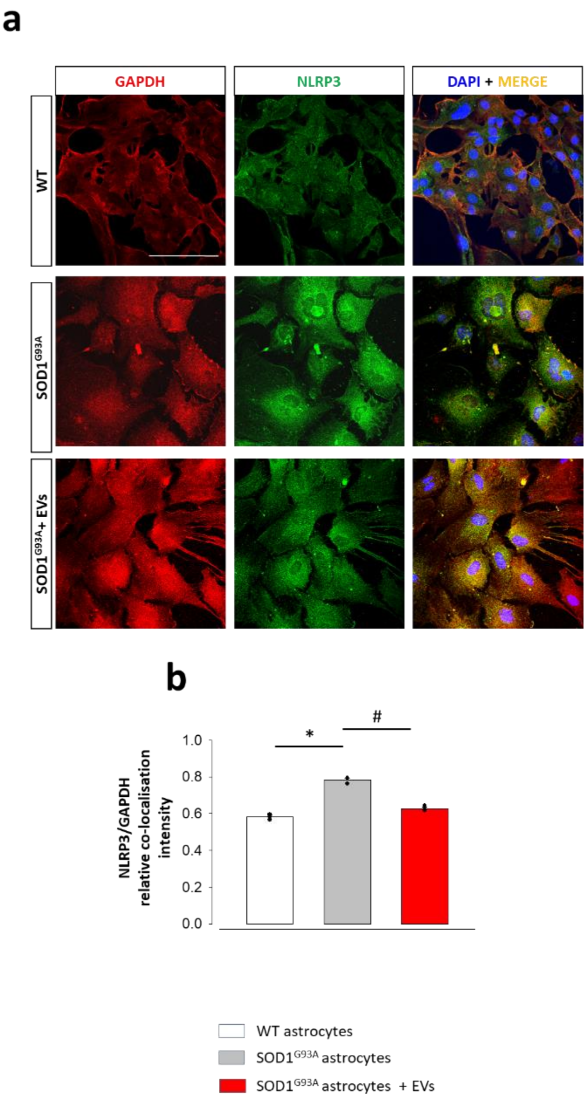

Supplementary figure 5. MSC-derived EVs reduce the NLRP3-inflammasome in astrocytes from the spinal cord of adult SOD1G93A mice.
(**a**) Representative confocal microscopy immunocytochemical images for GAPDH (red fluorescenmce) and NLRP3 (green fluorescence), and DAPI (blue fluorescence), in WT, untreated SOD1G93A astrocytes and SOD1G93A astrocytes treated with EVs. Scale bar: 100 µm. (**b**) Quantitative representation of the relative fluorescence intensity (see figure 2 for details) for NLRP3, in WT astrocytes, untreated SOD1G93A astrocytes and EVs-treated SOD1G93A astrocytes. Data are presented as means ± SEM of N=3 independent experiments, run in triplicate; statistical significance for $p<0.05$ at least (*$p<0.001$ vs. WT and #$p<0.001$ vs. SOD1G93A astrocytes; $F(2,6)=139.284$; one-way ANOVA, followed by Bonferroni post hoc test).

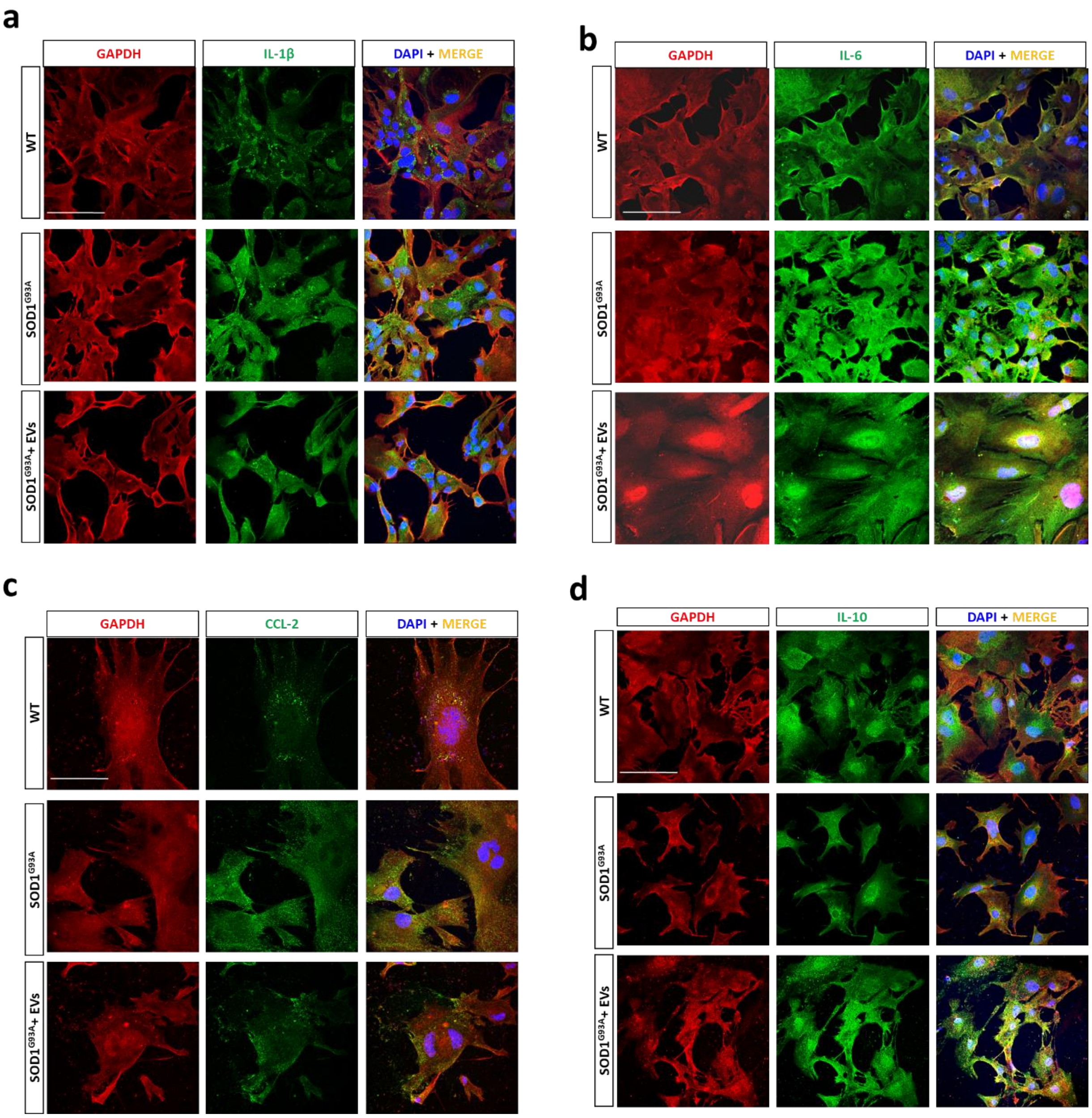

Supplementary Figure 6. MSC-derived EVs reduce the expression of pro-inflammatory cytokines in astrocytes of adult SOD1G93A mice.
Representative confocal microscopy immunocytochemical images for GAPDH (red fluorescence), (**a**) IL-1β, (**b**) IL-6, (**c**) CCL2, and (**d**) IL-10 (green fluorescence), and DAPI (blue fluorescence) in WT astrocytes, SOD1G93A astrocytes and EVs-treated SOD1G93A astrocytes. Scale bar: 100 μm.

Supplementary table 1.
Details of fibroblast donors.

Sample	Type of ALS	Sex	Age at biopsy collection (years)	Ethnicity	Onset to death (months)
CTR155	Non-ALS control	M	40	Caucasian	-
Pat78	C9orf72	M	66	Caucasian	31.7
Pat183	C9orf72	M	50	Caucasian	27
Pat100	SOD1 (A4V mutation)	F	N/A	Caucasian	-
Pat102	SOD1 (A4V mutation)	F	N/A	Caucasian	-

Supplementary table 2.
List and sequence of the synthetic mimics used for mouse and human astrocyte transfections.

Mouse miR mimics
miR-467f 5'-AUAUACACACACACACCUACA-3'
miR-466q5'-GUGCACACACACACAUACGU-3'
miR-466m-5p 5'-UGUGUGCAUGUGCAUGUGUGUAU-3'
miR-466i-3p 5'-AUACACACACACACAUACACACUA-3'
miR-466i-5p 5'-UGUGUGUGUGUGUGUGUGUG-3'
miR-467g 5'-UAUACAUACACACACACAUAUAU-3'
miR-3082-5p 5'-GACAGAGUGUGUGUGUCUGUGU-3'
miR-5126 5'-GCGGGCGGGGCCGGGGGCGGGG-3'
miR-669c-3p 5'-UACACACACACACACACAAGUAAA-3'
Human miR mimic
miR-29b-3p 5'-UAGCACCAUUUGAAAUCAGUGUU-3'

Supplementary table 3.
Details of antibodies

PRIMARY ANTIBODIES	WORKING DILUTION	PRODUCER AND CATALOG NUMBER
rabbit polyclonal anti-Glial fibrillary acid protein (GFAP) antibody	1:1000	Sigma-Aldrich, Cat# G4546
mouse monoclonal anti-Vimentin antibody	1:1000	Sigma-Aldrich, Cat# V2258
mouse monoclonal anti-S100β antibody	1:500	Chemicon International, Cat# MAB079-1
rabbit recombinant monoclonal anti-NLRP3 antibody	1:100	Abcam, Cat# ab210491
rabbit polyclonal anti-Nrf2 antibody	1:500	Abcam, Cat# ab31163
goat polyclonal anti-IL1β antibody	1:1000	Sigma-Aldrich, Cat# I3767
goat polyclonal anti-TNF-α antibody	1:300	Sigma-Aldrich, Cat# T0938
chicken polyclonal anti-IL-6 antibody	1:500	Sigma-Aldrich, Cat#GW22495
rat monoclonal anti-monocyte chemotactic protein-1 (MCP-1) antibody	1:500	Abcam, Cat# ab8101
rabbit polyclonal anti-IL-10 antibody	1:500	Abcam, Cat# ab996995
monoclonal anti-glyceraldehyde-3-phosphate dehydrogenase (GAPDH) antibody	1:1000	Sigma-Aldrich, Cat# G8795
chicken polyclonal anti-GAPDH antibody	1:1000	Abcam, Cat# ab83957
**SECONDARY ANTIBODIES**		
donkey anti-rabbit Alexa Fluor A488-conjugated	1:3000	Thermo Fisher Scientific, Cat# R37118
donkey anti-mouse Alexa Fluor A488-conjugated		Thermo Fisher Scientific, Cat# A21202
donkey anti-goat Alexa Fluor A488-conjugated		Thermo Fisher Scientific, Cat# A11055
goat anti-chicken Alexa Fluor A488-conjugated		Thermo Fisher Scientific, Cat# A11039
donkey anti-mouse Alexa Fluor A467-conjugated		Thermo Fisher Scientific, Cat# A31571
goat anti-chicken Alexa Fluor A467-conjugated		Thermo Fisher Scientific, Cat# A21449

Supplementary table 4.
Percentage of variation of GFAP, TNF-α and IL-1β expression in SOD1^{G93A} mouse-derived astrocytes after 48h transfection with single miRNA synthetic mimics vs. untreated SOD1^{G93A} astrocytes.

	G93A +Scramble	G93A +466q	G93A +467f	G93A +466m-5p	G93A +466i-5p	G93A +466i-3p	G93A +467g	G93A +5126	G93A +669c-3p	G93A +3082-5p
**GFAP**	-10%± 5.6	-40% ± 0.9	-57%±2.7	-68% ± 4.1	-80% ± 2.8	-54% ±3.0	-22% ± 2.7	-18% ± 1.8	-65% ± 2.4	-58% ± 2.4
**TNF-α**	-11% ±1.3	-97%± 1.5	-97%± 1.8	-100%± 2.4	-100%± 1.9	-113%± 3.0	-22% ± 2.5	-29%± 1.4	-110% ± 2.5	-97% ± 2.7
**IL-1β**	-4%± 2.8	-68% ± 4.2	-68% ± 4.2	-56% ± 1.7	-75% ± 2.9	-69% ± 5.0	-19% ± 3.5	-20% ± 2.1	-74% ± 2.7	-57% ± 2.9

Supplementary table 5.
List of the up-regulated miRNAs detected in human MSCs (hMSCs) primed with IFN-γ; the microarray analysis (LC-Science) has been performed in 3 independent human MSCs batches, three runs for each single batch.

		**hMSC**		**hMSC+IFN**		
**Reporter Name**	**p-value**	**Mean**	**StDev**	**Mean**	**StDev**	**Log2 (G2/G1)**
hsa-miR-27a-3p	7.25E-02	645	99	841	35	-0.38
hsa-miR-29b-3p	8.96E-02	1203	749	2431	388	-1.01
hsa-miR-7150	2.98E-02	24	4	33	2	-0.45

Supplementary table 5.
List of the up-regulated miRNAs detected in human MSCs (hMSCs) primed with IFN-γ; the microarray analysis (LC-Science) has been performed in 3 independent human MSCs batches, three runs for each single batch.

		**hMSC**		**hMSC+IFN**		
**Reporter Name**	**p-value**	**Mean**	**StDev**	**Mean**	**StDev**	**Log2 (G2/G1)**
hsa-miR-27a-3p	7.25E-02	645	99	841	35	-0.38
hsa-miR-29b-3p	8.96E-02	1203	749	2431	388	-1.01
hsa-miR-7150	2.98E-02	24	4	33	2	-0.45
